# Process accident prediction using Bayesian network based on IT2Fs and Z-number: A case study of spherical tanks

**DOI:** 10.1371/journal.pone.0307883

**Published:** 2024-08-29

**Authors:** Mostafa Mirzaei Aliabadi, Rouzbeh Abbassi, Omid Kalatpour, Omran Ahmadi, Vahid Ahmadi Moshiran

**Affiliations:** 1 Center of Excellence for Occupational Health, Occupational Health and Safety Research Center, School of Public Health, Hamadan University of Medical Sciences, Hamadan, Iran; 2 School of Engineering, Faculty of Science and Engineering, Macquarie University, Sydney, NSW, Australia; 3 Department of Occupational Health and Safety, Faculty of Medical Science, Tarbiat Modares University, Tehran, Iran; Istanbul University: Istanbul Universitesi, TÜRKIYE

## Abstract

This study aimed to propose a novel method for dynamic risk assessment using a Bayesian network (BN) based on fuzzy data to decrease uncertainty compared to traditional methods by integrating Interval Type-2 Fuzzy Sets (IT2FS) and Z-numbers. A bow-tie diagram was constructed by employing the System Hazard Identification, Prediction, and Prevention (SHIPP) approach, the Top Event Fault Tree, and the Barriers Failure Fault Tree. The experts then provided their opinions and confidence levels on the prior probabilities of the basic events, which were then quantified utilizing the IT2FS and combined using the Z-number to reduce the uncertainty of the prior probability. The posterior probability of the critical basic events (CBEs) was obtained using the beta distribution based on recorded data on their requirements and failure rates over five years. This information was then fed into the BN. Updating the BN allowed calculating the posterior probability of barrier failure and consequences. Spherical tanks were used as a case study to demonstrate and confirm the significant benefits of the methodology. The results indicated that the overall posterior probability of Consequences after the failure probability of barriers displayed an upward trend over the 5-year period. This rise in IT2FS-Z calculation outcomes exhibited a shallower slope compared to the IT2FS mode, attributed to the impact of experts’ confidence levels in the IT2FS-Z mode. These differences became more evident by considering the 10^−4^ variance compared to the 10^−5^. This study offers industry managers a more comprehensive and reliable understanding of achieving the most effective accident prevention performance.

## 1. Introduction

Spherical tanks currently have a significant function in the oil and gas industries [1]. They serve as a widely used storage option for various gases and fluids, like LPG (liquefied petroleum gas) and LNG (liquefied natural gas), owing to the advantageous characteristics linked to their form [2, 3]. However, in recent years, as this type of tank has increased in various sectors, several accidents have been involving them, including fires and explosions, resulting in significant human and financial losses [1, 4]. Here are some accidents related to spherical tanks in the past years. In 1984, a spherical LPG tank explosion in Mexico City devastated 270 houses spread across an area measuring 100,000 square meters. Tragically, the accident claimed 500 lives and caused injuries to an additional 4,000 individuals [5–7]. Similarly, at a Texas refinery in 1978, a BLEVE incident led to the rupture and explosion of three spherical tanks, five horizontal tanks, and four vertical tanks, resulting in the loss of at least seven lives [8]. 1966 the Feyzin refinery accident in France caused 18 fatalities and 81 injuries [8, 9]. Furthermore, in Montreal, Canada 1957, a defective level gauge led to a fatality caused by BLEVE spherical tanks [8]. Lastly, the Visakhapatnam refinery incident in India in 1997 resulted in 56 deaths and approximately $15 million in damages [10].

These events indicate that despite the low probability of spherical tank accidents, the potential consequences can be significant. Moreover, the absence of a proportional risk assessment framework may contribute to disastrous accidents [2, 11]. As a result, it is crucial to develop a dynamic risk assessment method that can take into account changes in the process and help prevent accidents related to spherical tanks [2, 12]. Dynamic risk assessment adjusts the initial risk rating based on factors such as the reliability of safety systems, maintenance and inspection procedures, human behavior, and operational processes [11, 13].

The development of studies on dynamic risk assessment in process facilities is ongoing. Zarei et al. (2021) employed a BN using the best-worst method (BWM) and D-number theory in the dynamic risk assessment of hydrogen infrastructures, aiming to minimize uncertainty in determining the prior probability of BEs [14]. Using the temporal evolution of escalation vectors with the Dynamic Bayesian Network (DBN) approach was the method used by Zeng et al. to estimate the exact dynamic probabilities of domino effects in chemical industrial areas [15]. Kamil et al. (2019) presented the Stochastic Petri-net model for studying the probability and propagation pattern of domino events to evaluate the dynamic risk associated with domino effects [16]. Luan et al. (2023) analyzed the factors influencing the dynamic risk assessment for road tanker accidents using a fuzzy Bayesian network (FBN) and binary logistic regression model combined with a bow-tie model [17]. In other research, Bhandari et al. (2016) combined a dynamic risk-based strategy with a maintenance-optimizing technique to develop a maintenance plan [18]. Zhou et al. (2023) conducted a risk assessment study of an oil storage tank using the bowtie model. They used expert judgment and fuzzy sets to obtain prior probabilities of basic incidents. They then used new evidence and posterior probabilities of basic incidents to calculate posterior probabilities of storage tank consequences [19]. As a quantitative risk assessment technique to assess safety at each stage of the accident sequence analysis, Rathnayaka et al. introduced SHIPP (System Hazard Identification, Prediction, and Prevention) [20, 21]. This method presents a structured approach to recognize, evaluate, and model accidents during processes, foresee their probability and implement measures to prevent them [22]. By comprehending accidents within intricate systems, this model transcends the constraints of traditional accident models. It estimates the likelihood of accidents happening based on historical accident data and has the ability to adapt the probability of accidents occurring [2]. To connect cause and effect in this model, the fault and event tree are combined, and Bayesian inference is employed to deal with data uncertainty [22].

The usefulness of this technique for modeling accidents has been supported by numerous studies in the process industries. Pouyakian et al. (2021) used the combination of SHIPP and HAZOP techniques to investigate managerial, organizational, human, and process factors to provide a thorough approach based on FBN for modeling and reducing the uncertainty of parameters as well as an accurate analysis of the risk of release of hazardous substances in Storage tanks with a floating roof [23]. Sarvestani et al. (2021) conducted a study using the MIMAH and SHIPP methods to model the occurrence process of accidents in LPG storage tanks [2]. In another study, the combination of SHIPP with HFACS, FBWM, and FBN methods was offered as a novel model for overcoming the limitations of other studies in the analysis of human and organizational aspects in the occurrence of accidents related to ethylene storage tanks and their consequences [22]. Due to the accident scenario being displayed from causes to consequences, BT is a popular technique in the risk assessment of process systems. This model can help determine and evaluate the root cause of system failure and its potential consequences. It improves FT and ET’s capabilities by combining them into a single approach [24, 25]. However, due to the static nature of FTA and ETA in its structure, it is limited in the dynamic risk assessment. Additionally, BT cannot demonstrate conditional reliance. These limitations are overcome by the BN [26]. Moreover, studies have demonstrated the flexible framework of BN in effectively analyzing various accident scenarios for dynamic safety assessment [26–28]. Khakzad et al. (2013) presented an approach that combines the bowtie technique with the Bayesian network framework. They aimed to create a dynamic tool that effectively addresses uncertainties by leveraging the Bayes theorem [26, 29]. BN analysis is favored due to several critical factors compared to traditional techniques like FT and ET. One primary advantage lies in its capacity to simulate intricate systems while diminishing parametric uncertainty by the assimilation of additional evidence. Moreover, its user-friendly and concise graphical approach is highly valued. Furthermore, BN enables probability updating and sequential learning [18, 30].

Zerouali et al. (2019) used FBN to predict the risk of storage tank fire and explosion. They mapped the Bowtie in BN to quantify the probabilities of events [31]. Sun et al. (2022) addressed the challenge of safety assessment in complex, dynamic process systems. They propose a method that combines Catastrophe Theory for quantifying disruption intensity with a Dynamic Bayesian Network (DBN) to model system performance response. This framework offers a resilience metric for evaluating a system’s ability to absorb, adapt, and recover from disruptions [28]. Yazdi and Kabir (2018) proposed a methodology for process systems risk assessment to Highlight the limitations of traditional QRA. Their approach integrated Bayesian Networks, Fuzzy Set Theory, and Evidence Theory to address data uncertainty, expert opinion subjectivity, and model limitations [32].

The data quantity and knowledge limitations result in vague and imprecise information used in the suggested frameworks, irrespective of whether BN is utilized. Employing fuzzy logic can resolve this imprecision effectively [33]. It is challenging to use a comprehensive database to cover all systems since the probability of a basic event occurring in various industries is dependent on local conditions. As a result, the primary data inputs for probability estimation are expert judgments. But these opinions frequently contain ambiguity and imprecision. Thus, Zadeh presented a Type-I fuzzy set theory to solve this problem [19, 34].

Several research used FTA with the Fuzzy type-1 set in various industries [35–39]. However, this quantification approach based on experts’ opinions cannot show the uncertainties related to the membership functions [37, 40]. Therefore, a type two fuzzy set (T2FS) is developed for more accurate risk quantification [24, 41].

Different individuals assign distinct interpretations to identical words; hence, Zadeh (1975) introduced T2FSs to capture experts’ subjective and imprecise judgment in real-world operations [41]. In other words, each element of T2FSs, unlike a T1FS, which presents the degree of membership as a crisp number in [0,1], is an interval in [0, 1] [42]. Three-dimensional T2FS membership functions provide more degrees of freedom for dealing with uncertainties and direct modeling [43].

Mendel and John (2002) put forward the interval type-2 fuzzy set (IT2FS) as a specific form of T2FSs, which has gained popularity due to its simplicity and widespread application [44]. In some studies, different methods like IT2FTOPSIS, IT2FAHP, IT2FDEMATEL, IT2FVIKOR, IT2FFMEA, and T2FS with D-S evidence theory were used [45–49]. Z-number is a novel idea a more remarkable ability to express human understanding. It has a simple framework with reliability and restrictions that makes it easy to express and manage the confidence of uncertain information [50].

This study proposes a novel approach to address these limitations. It leverages Interval Type-2 Fuzzy Sets (IT2FS) and Z-numbers within a Bayesian Network framework. With the IT2FS combination and the Z-number, this approach provides reliable quantitative values for input for calculations in the Bayesian network and beta distribution, increasing the reliability of predicting process accidents.

IT2FS and Z-numbers (IT2FS-Z) integration within a BN framework has received limited attention in the existing literature. By incorporating Z-numbers, we move beyond traditional fuzzy logic approaches (Level 2) and exact number calculations (Level 1) to a more nuanced representation of uncertainty that is closer to real-world human decision-making (Level 3) [51, 52]. This allows for a more reliable and realistic risk assessment, particularly in scenarios with limited data and subjective expert opinions.

After reviewing previous research, only two studies were found that used the combination of z-number and IT2FS. In one of these studies, Zamri et al. used Z-number, IT2FS, and TOPSIS to deal with decision uncertainty [49]. In another study, Azman et al. improved IT2FVIKOR with Z-numbers to determine the best strategy for water supply security in Malaysia [53]. These researchers attempted to use the IT2FS and Z number (IT2FS-Z) to reduce decision uncertainty. However, no studies using the beta distribution based on the prior probability obtained using IT2FS-Z to calculate the posterior probability of barrier failure and consequences were identified.

In this regard, the present study aims to provide a method to improve the confidence level in dynamic risk assessment by combining IT2FS-Z and beta distribution in the BN platform to reduce the uncertainty in determining the posterior probability of accident occurrence.

## 2. Theoretical background

### 2.1. Interval type-2 fuzzy sets

The definitions and arithmetic operations of BN, Z-numbers, power average operators, and IT2FS are introduced in this section. It is worth noting that a trapezoidal membership function (TrMF) was employed throughout the study since it can yield results that are more accurate than those produced by triangular MF [54].

#### Definition 1 [55]

The following is the expression for a T2FS with the membership function (MF), $\mu_{\tilde{\tilde{A}}}$ (x, μ), where x ∈ X:

$$\tilde{\tilde{A}}=\{\left((x,u),\mu_{\tilde{\tilde{A}}}(x,u))|\forall x\in X,\forall_{u}\in J_{x}\subseteq [0,1]\right\}$$ (1)

where *J*_*x*_ denotes an interval in [0, 1] and $0\leq \mu_{\tilde{\tilde{A}}}(x,u)\leq 1$. Moreover, $\tilde{\tilde{A}}$ *can also be represented as* the

following form:

A˜˜=∫x∈X∫μ∈JxμA˜˜(x,u)(x,u),Jx⊆[0,1] (2)

where ∫ ∫ indicates the union of all valid x and u. The primary and secondary variables of $\tilde{\tilde{A}}$ are x and u, respectively.

#### Definition 2

Interval type-2 fuzzy sets (IT2FS), a particular instance of T2FS, are what set $\tilde{\tilde{A}}$ is known as when all $\mu_{\tilde{\tilde{A}}}(x,u)$ = 1. IT2FS $\tilde{\tilde{A}}$ can be expressed as follows using Eq (2):

A˜˜=∫x∈X∫u∈Jx1(x,u) (3)

#### Definition 3

**Fig 1:**
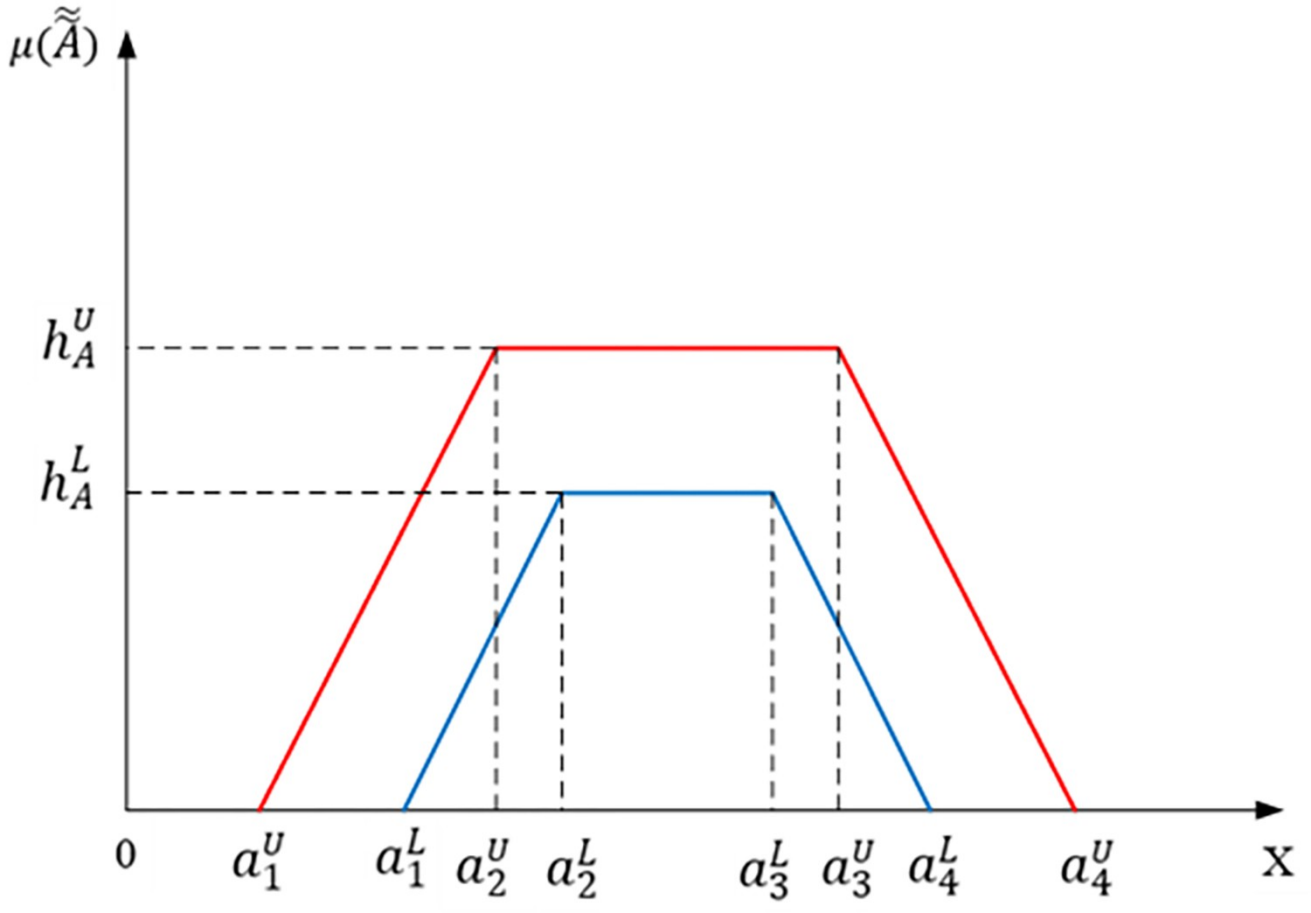
The membership functions of the IT2FSs.

An IT2FS has two membership functions, the top and lower of which are type-1 membership functions. A trapezoidal IT2FS $\tilde{\tilde{A}}$ as provided in Eq 4 is depicted in Fig 1 [56].


$$\tilde{\tilde{A}}=[{\tilde{A}}^{U},{\tilde{A}}^{L}]=[\left(a_{1}^{U},a_{2}^{U},a_{3}^{U},a_{4}^{U};h_{A}^{U}),(a_{1}^{L},a_{2}^{L},a_{3}^{L},a_{4}^{L};h_{A}^{L}\right)]$$ (4)

Where $a_{1}^{U},a_{2}^{U},a_{3}^{U},a_{4}^{U}$ and $a_{1}^{L},a_{2}^{L},a_{3}^{L},a_{4}^{L}$ represent the reference points of the IT2FS $\tilde{\tilde{A}}$ in which ${\tilde{A}}^{U}$ and ${\tilde{A}}^{L}$ are the T1FS, with $a_{1}^{U}\leq a_{2}^{U}\leq a_{3}^{U}\leq a_{4}^{U},a_{1}^{L}\leq a_{2}^{L}\leq a_{3}^{L}\leq a_{4}^{L},a_{1}^{U}\leq a_{1}^{L}\;and\;a_{4}^{L}\leq a_{4}^{U}\;$. Where the heights of the lower membership function ($h_{A}^{L}$) and the upper membership function ($h_{A}^{U}$) of IT2FS, $\tilde{\tilde{A}}$, respectively, are shown in Fig 1.

#### Definition 4

Let there be two IT2FS, ${\;\tilde{\tilde{A}}}_{1}=[\left(a_{11}^{U},a_{12}^{U},a_{13}^{U},a_{14}^{U};h_{1A}^{U}),(a_{11}^{L},a_{12}^{L},a_{13}^{L},a_{14}^{L};h_{1A}^{L}\right)]$ and ${\;\tilde{\tilde{A}}}_{2}=[\left(a_{21}^{U},a_{22}^{U},a_{23}^{U},a_{24}^{U};h_{2A}^{U}),(a_{21}^{L},a_{22}^{L},a_{23}^{L},a_{24}^{L};h_{2A}^{L}\right)]$. Then, the following describes how to perform calculations between trapezoidal IT2FS.

Addition operation:

A˜˜1⊕A˜˜2=[(a11U+a21U,a12U+a22U,a13U+a23U,a14U+a24U;min{h1AU,h2AU})(a11L+a21L,a12L+a22L,a13L+a23L,a14L+a24L;min{h1AL,h2AL})] (5)

Subtraction operation:

A˜˜1⊖A˜˜2=[(a11U−a21U,a12U−a22U,a13U−a23U,a14U−a24U;min{h1AU,h2AU})(a11L−a21L,a12L−a22L,a13L+a23L,a14L−a24L;min{h1AL,h2AL})] (6)

Multiplication operation:

A˜˜1⊗A˜˜2=[(a11U×a21U,a12U×a22U,a13U×a23U,a14U×a24U;min{h1AU,h2AU})(a11L×a21L,a12L×a22L,a13L×a23L,a14L×a24L;min{h1AL,h2AL})] (7)

Multiplication with a scalar k > 0:

k.A˜˜1=[(k×a11U,k×a12U,k×a13U,k×a14U;h1AU)(k×a11L,k×a12L,k×a13L,k×a14L;h1AL)] (8)

Power operation:

(A˜˜1)α=[(a11U)α,(a12U)α,(a13U)α,(a14U)α;h1AU)((a11L)α,(a12L)α,(a13L)α,(a14L)α;h1AL)] (9)

d is distance between ${\;\tilde{\tilde{A}}}_{1}$ and ${\;\tilde{\tilde{A}}}_{2}$ as following [57]:

$$d({\;\tilde{\tilde{A}}}_{1},{\;\tilde{\tilde{A}}}_{2})=\left|R_{d}({\tilde{\tilde{A}}}_{1},\tilde{\tilde{1}})-R_{d}({\tilde{\tilde{A}}}_{2},\tilde{\tilde{1}})\right|$$ (10)

where $\tilde{\tilde{1}}$ = [(1, 1, 1, 1; 1), (1, 1, 1, 1; 1)], and

$$R_{d}({\tilde{\tilde{A}}}_{1},\tilde{\tilde{1}})=\frac{1}{2.h_{1A}^{U}.h_{1A}^{L}}\left[h_{1A}^{U}.(a_{14}^{L}-a_{13}^{L}-a_{14}^{U}+a_{13}^{U})\right]-\frac{1}{2.h_{1A}^{U}.h_{1A}^{L}}\left[h_{1A}^{L}.(0.5({(a}_{12}^{U}-a_{11}^{U}-a_{12}^{L}+a_{11}^{L})-(a_{14}^{U}-a_{13}^{U}-a_{12}^{U}+a_{11}^{U}))\right]+1-a_{14}^{U}-0.5(a_{11}^{U}-a_{11}^{L}+a_{14}^{L}-a_{14}^{U})$$ (11)

### 2.2. The power average operator

The Power Average (PA) operator is widely recognized as an effective method for combining individual preferences to derive group preference values that accurately represent the correlation between risk assessments. It is described as follows.

#### Definition 6

The PA operator, with a dimension of n, is a mapping (PA) that takes Rn to R. This can be represented by Eq 12 [58].


$$PA(a_{1}\;a_{2},\ldots ,a_{n}\;)=\frac{\;\sum_{i=1\;}^{n}(1+T(a_{i}))a_{i}}{\sum_{i=1}^{n}\left(1+T(a_{i})\right)}$$ (12)

Where

$$T(a_{i})=\sum_{j=1,j\neq i}^{n}\sup\left(a_{i},a_{j}\right),i\in N,$$ (13)

The following are the characteristics of the sup(*a*_*i*_, *a*_*j*_), which expresses the degree that *a*_*j*_ supports the *a*_*i*_:

1. sup(*a*_*i*_, *a*_*j*_) ∈ [0, 1]
2. sup(*a*_*i*_, *a*_*j*_) = sup(*a*_*j*_, *a*_*i*_)
3. sup(*a*_*i*_, *a*_*j*_) ≥ Sup π (x, y), if d (*a*_*i*_, *a*_*j*_) < d (x, y)

The closer two values are to one another under condition three above, the more they support one another. According to Son et al. [59], the support measure (Sup) is a similarity measure. Consequently, the equation provided below can be utilized to earn *a*_*j*_’s support for *a*_*i*_:

$$\sup\left(a_{i},a_{j}\right)=1-d(a_{i},a_{j})$$ (14)

### 2.3. Z-number

**Definition 5**. Z-number denoted as Z = ($\tilde{\tilde{A}},\tilde{\tilde{B}}$), comprises two components. A restriction on the values is mentioned for the first component, $\tilde{\tilde{A}}$. The second component, $\tilde{\tilde{B}}$, provides a measure of the first component’s reliability. Generally, $\tilde{\tilde{A}}$ and $\tilde{\tilde{B}}$ are subjective and can be conveyed using natural language [60, 61].

**Fig 2:**
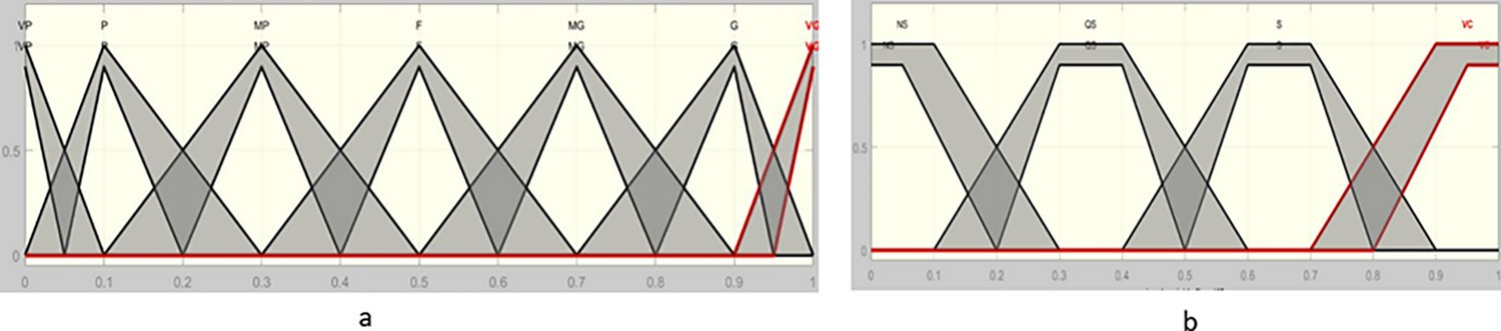
Interval type 2 trapezoid fuzzy membership functions. (a: related to opinion b: related to confidence).

For example, if the expert’s opinion about an event is "Medium High" and her level of confidence is "Relatively sure," it can be written as a *Z*-number as follows: *Z* = (Medium High, relatively sure). According to Table 1, this state will be quantitatively Z = [((0.5, 0.7, 0.7, 0.9; 1), (0.6, 0.7, 0.7, 0.8; 0.9)), ((0.1, 0.3, 0.4, 0.6; 1), (0.2, 0.3, 0.4, 0.5; 0.9))]. $\tilde{\tilde{A}}$ and $\tilde{\tilde{B}}$ are assumed as trapezoidal fuzzy numbers, as defined in Fig 2. The mathematical definition of $\tilde{\tilde{A}}$ and $\tilde{\tilde{B}}$ is similar to Eq 1. $\tilde{\tilde{B}}$ can be converted into a crisp value by means of Eq 15 as follows [43, 51].

α=∫x∈X∫μ∈JxμB˜˜(x,u)(x,u)xdx∫x∈X∫μ∈JxμB˜˜(x,u)(x,u)dx (15)

Where *α* stands for the weight of the second portion $\tilde{\tilde{B}}$ and $\mu_{\tilde{\tilde{B}}}(x,u)$ for the degree to which *x* ∈ *X* is dependent on $\tilde{\tilde{B}}$. Then, *α* can be added to the initial element $\tilde{\tilde{A}}$ using Eq 16. (51).

$${\tilde{\tilde{z}}}^{\alpha }=\{\left(x,\mu_{{\tilde{\tilde{B}}}^{\alpha }}\;\right)|\mu_{{\tilde{\tilde{B}}}^{\alpha }}(x,u)=\alpha \mu_{{\tilde{\tilde{B}}}^{\alpha }}(x,u),x\in (0,1)\}$$ (16)

where $\mu_{{\tilde{\tilde{B}}}^{\alpha }}$ is a representation of the degree to which *x* ∈ *X* is dependent on ${\tilde{\tilde{B}}}^{\alpha }$. Consequently, it was possible to acquire the rules for converting Z-number linguistic variables to IT2F by combining the linguistic variables.

**Table 1:** Linguistic terms and their corresponding IT2FSs [51].

Linguistic terms of opinion	Interval type-2 trapezoid fuzzy sets	Linguistic terms of confidence level	Interval type-2 trapezoid fuzzy sets
**Very Low (VL)**	[(0, 0, 0, 0.1; 1), (0, 0, 0, 0.05; 0.9)]	**Not sure (NS)**	[(0, 0, 0.1, 0.3; 1), (0, 0, 0.05, 0.2; 0.9)]
**Low (L)**	[(0, 0.1, 0.1, 0.3; 1), (0.05, 0.1, 0.1, 0.2; 0.9)]	**Relatively sure (QS)**	[(0.1, 0.3, 0.4, 0.6; 1), (0.2, 0.3, 0.4, 0.5; 0.9)]
**Medium Low (ML)**	[(0.1, 0.3, 0.3, 0.5; 1), (0.2, 0.3, 0.3, 0.4; 0.9)]	**Sure (S)**	[(0.4, 0.6, 0.7, 0.9; 1), (0.5, 0.6, 0.7, 0.8; 0.9)]
**Medium (M)**	[(0.3, 0.5, 0.5, 0.7; 1), (0.4, 0.5, 0.5, 0.6; 0.9)]	**Very confident (VC)**	[(0.7, 0.9, 1, 1; 1), (0.8, 0.95, 1, 1; 0.9)]
**Medium High (MH)**	[(0.5, 0.7, 0.7, 0.9; 1), (0.6, 0.7, 0.7, 0.8; 0.9)]		
**High (H)**	[(0.7, 0.9, 0.9, 1; 1), (0.8, 0.9, 0.9, 0.95; 0.9)]		
**Very High (VH)**	[(0.9, 1, 1, 1; 1), (0.95, 1, 1, 1; 0.9)]		

In the final step, it is suggested that the asymmetrical IT2F number (based on experts’ weighted views) be converted to the symmetrical IT2F number Eq 17 [62].

$${\tilde{\tilde{z}}}^{\prime }=\left\{\left(x,\mu_{{\tilde{\tilde{Z}}}^{\prime }}\;\right)|\mu_{{\tilde{\tilde{Z}}}^{\prime }}(x,u)=\mu_{\tilde{\tilde{A}}}(\frac{x}{\sqrt{\alpha }}),x\in [0,1]\right\}$$ (17)

Where $\mu_{{\tilde{\tilde{z}}}^{\prime }}(x)$ can be defined as fallow:

$$\mu_{{\tilde{\tilde{z}}}^{\prime }}(x,u)=\mu_{\tilde{\tilde{A}}}(\frac{x}{\sqrt{\alpha }}),\;x\in \sqrt{\alpha }X$$ (18)

### 2.4. Bayesian network

#### Definition 7

Conditional Probability Tables (CPTs) are employed in the BN model to determine the probability of BEs. Using these tables, the probability of an intermediate node can be calculated based on its conditional dependencies with related root nodes.

The common probability distribution of a collection of variables is determined in BN using Eq 18.

$$P(U)=\prod_{i=1}^{n}p(A_{i}|Pa(A_{i}))$$ (19)

Where Pa (Ai) denotes the parent set of Ai in the BN, and P (U) refers to the BN’s characteristics [63].

When new information (E evidence) is considered, BN updates the prior probability of occurrences using Bayes’ theorem to obtain the posterior probability. This new observation typically becomes accessible throughout the operational lifetime of a process, including the occurrence or nonoccurrence of primary events or accidents:

$$P(U|E)=\frac{P(U,E)}{P(E)}=\frac{P(U,E)}{\sum_{U}P(U,E)}$$ (20)

### 2.5. Beta distribution

#### Definition 8

The discrete value of the BE failure probabilities (FPs) can be explained by the mean (*μ*) of the distribution, where *α* and *β* are the factors defined as the success and failure of equipment in response to demand in the beta distribution. This means that the discrete value of the FPs can be expressed as a continuous value.


$$FP=\mu =\frac{\alpha }{\alpha +\beta }$$ (21)

Suppose the only information available regarding the reliability or likelihood of BEs is the discrete value of the FP. In that case, the degree of certainty (FP = *μ*), which measures how spread out the distribution is around it, needs to be determined by the variance (VAR) of the distribution:

$$VAR=\frac{\alpha .\beta }{{\left(\alpha +\beta \right)}^{2}.(\alpha +\beta +1)}$$ (22)

When there is a higher variance in the data, it becomes less reliable and more sensitive to changes in the following data.

By obtaining FP values of BEs based on experts’ opinions and the amount of variance from previous studies [64], it is possible to calculate the initial value of *α* and *β* in year zero, represented by *α*_0_ and *β*_0_.

$$\beta_{0}=\left(\frac{FP.(1-FP)-VAR}{VAR}\right).(1-FP)$$ (23)

$$\alpha_{0}=\frac{FP\times \beta_{0}}{\left(1-FP\right)}$$ (24)

Where VAR was considered 10^−4^ and 10^−5^ so that scores could be compared. The posterior beta distribution represents the updated FP of each BEs (Eq 25).

$${FP}_{1}=\frac{\alpha_{1}}{\alpha_{1}+\beta_{1}}$$ (25)

$$\alpha_{1}=\alpha_{0}+F$$ (26)

$$\beta_{1}=\beta_{0}+S$$ (27)

Where F and S are the numbers of failures and successes of BEs during the expected lifetime of the system, respectively. The indices for the prior and posterior distributions are given by the numbers 0 and 1, respectively.

## 3. Proposed methodology for assessing dynamic risks

**Fig 3:**
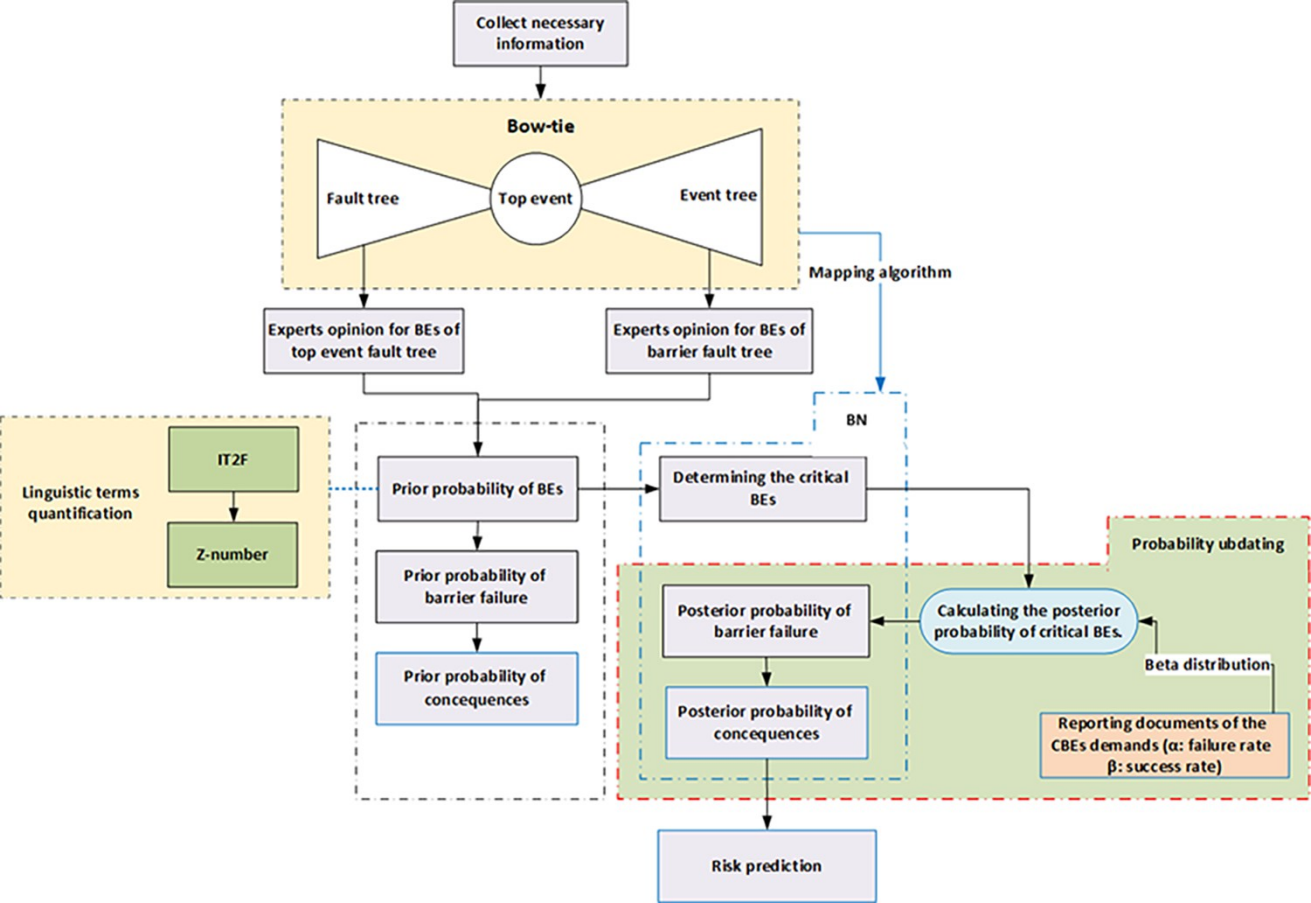
Work flow diagram of proposed method.

In this section, the steps of implementing the research method are explained. Fig 3 illustrates the step-by-step process of the proposed methodology.

### 3.1. Hazards identification

#### 3.1.1. Combining the bow-tie with the SHIPP approach

**Fig 4:**
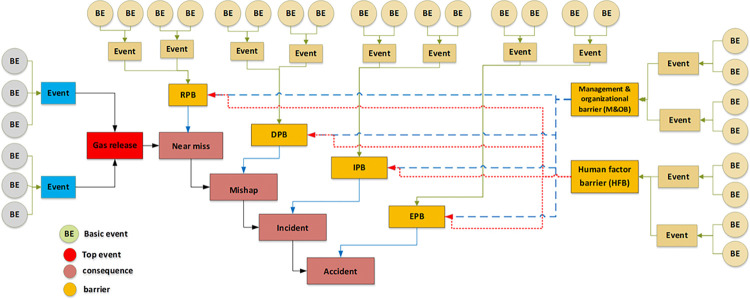
The link between barriers and how they affect the consequences based on the SHIPP theory.

In stage one, the Bes impacting the top event were identified, and a fault tree was also formed for the obstacles. Then, the bow-tie diagram was created using the SHIPP approach. The SHIPP approach follows a specific order and hierarchy of event consequences, which closely resemble the structural development of an event tree. The consequences are Respectively as follows. Near miss, mishap, incident, accident. In reality, the occurrence of an end event can potentially escalate in any sequence. For instance, a near miss could potentially turn into an accident (Fig 4) [65].

Safety barrier has been categorized in the SHIPP approach for accident prevention techniques after release factor; 1. Release Prevention Barrier (RPB), 2. Dispersion Prevention Barrier (DPB), 3. Ignition Prevention Barrier (IPB), 4. Escalation Prevention Barrier (EPB), 5. Human Factor Barrier (HFB), 6. Management and Organizational Barrier (MOB) [20].

The bow-tie diagram, which can be observed conceptually in Fig 4, was drawn based on this approach and considering the series of consequences, including near miss, mishap, incident, and accident. Fig 4 illustrates the improved predictive accuracy of presented model, clearly showing how it outperforms existing models during the simulated accident scenarios.

### 3.2. Aggregating stage

#### 3.2.1. Weight of expert judgment

Twenty experts’ opinions and confidence levels on the prior probability of BEs were obtained. Their linguistic terms were then quantified and combined using IT2FS and Z-number. It is important to note that the experts’ involvement was solely intended to assist the researchers in gaining a comprehensive understanding of the industrial process under investigation and to estimate the probability of BE occurrences and obstacle failure probabilities. Written informed consent was obtained from all twenty participating industry specialists before their involvement in the study, conducted between January 15^th^ and April 23^rd^, 2023. Ethical approval was obtained from the ethics committee of Hamadan University of Medical Sciences. Details of Ethical approval are available in the Acknowledgments section.

It is remarkable that when integrating the views of many experts, it is important to consider the weight of the experts in order to make the evaluation results more scientific and objective. The Linguistic terms given by them were employed for the probability of BEs occurrence and corresponding confidence level, which fall into (“VL”, “L”, “ML”, “M”, “MH”, “H”, “VH”) and (“NS”, “QS”, “S”, “VS”), respectively (Table 1). Table 2, presents data on the age(A), education level (EL), job title (JT), and related experience (RE) of each expert.

**Table 2:** Weighting scores of various experts [66].

Group	Classification	Score
**Job title**	Operator	1
	Technical	2
	Engineer	3
	Manager, Factory inspection	4
	Chief Engineer, Director	5
**Educational level**	High school	1
	Higher national diploma	2
	Bachelor	3
	Master	4
	PhD	5
**Related experience (year)**	≤ 5	1
	6 to 9	2
	10 to 19	3
	20 to 29	4
	≥ 30	5
**Age**	<30	1
	30 to 39	2
	40 to 49	3
	≥50	4

A score is assigned to each expert based on this information. Using the experts’ weight scores and weight factors, Eqs (28) and (29) calculate this score (Table 3).

**Table 3:** Calculation weighting factors for 20 experts.

Symbol	Experts	Job title	Educational degree	Years of related experience	Age	score	weight factor (Wi)
**E1**	Refining Engineer	3	3	1	1	8	0.0373832
**E2**	Head of HSE	4	4	3	3	14	0.0654206
**E3**	HSE Expert	3	3	1	2	9	0.0420561
**E4**	Non-Destructive Testing Expert	3	3	2	2	10	0.046729
**E5**	Control Room Supervisor	4	4	3	3	14	0.0654206
**E6**	Equipment quality control inspector	3	3	2	2	10	0.046729
**E7**	Process Safety Expert	3	4	2	2	11	0.0514019
**E8**	Maintenance technician	2	2	2	1	7	0.0327103
**E9**	Precision instrument expert	2	2	3	2	9	0.0420561
**E10**	Machinery and Equipment Safety Expert	3	3	3	3	12	0.0560748
**E11**	Chief of the fire department	4	4	3	4	15	0.0700935
**E12**	Technical inspection of tanks	4	4	2	2	12	0.0560748
**E13**	Refining Engineer	3	3	2	3	11	0.0514019
**E14**	Non-Operating Defense	4	4	3	3	14	0.0654206
**E15**	HSE Expert	3	3	2	2	10	0.046729
**E16**	Senior process design engineer	4	4	2	3	13	0.0607477
**E17**	Responsible for the electricity of the reservoirs	3	3	2	2	10	0.046729
**E18**	Maintenance technician	2	2	1	3	8	0.0373832
**E19**	Machinery and Equipment Safety Expert	3	3	1	2	9	0.0420561
**E20**	Process Safety Expert	3	3	1	1	8	0.0373832


$$\mathrm{The}\;\mathrm{weight}\;\mathrm{score}\;\mathrm{of}\;j^{th}\;\mathrm{Expert}=\mathrm{Score}\;\mathrm{of}\;A_{j}+\mathrm{Score}\;\mathrm{of}\;JT_{j}+\mathrm{Score}\;\mathrm{of}\;EL_{j}+\mathrm{Score}\;\mathrm{of}\;RE_{j}$$ (28)

$$W_{j}=\frac{\;The\;weight\;score\;of\;j^{th}\;Expert\;}{\overset{n}{\underset{j=1}{\sum }}\;The\;weight\;score\;of\;Experts\;}$$ (29)

#### 3.2.2. Expert opinion aggregation using the power average operator

Although widely used data aggregation methods like average, median, and mode, they sometimes fail to convey the complexity via the aggregated statistic. Considering the links between the variables being combined would help to make these integrating mechanisms intelligent [58].

Due to the experts’ varying experiences, knowledge, and preferences, the assessment information provided by the experts may vary throughout the entire assessment procedure. As a result, in this research, a group decision matrix was created using an aggregation method to take into consideration of individual assessment data. By applying the power average operator (PA) in the aggregation process in risk assessment, lower weight is given to bigger or smaller evaluation values. Therefore, the opinions of the group’s experts become closer and create more reliable results. Therefore, the issue of combining expert views is solved using the PA-IT2FS-Z operator which is based on the PA operator theory [67].

The fuzzy aggregate outcome of the expert views is represented by R_i_ (i = 1, 2, 3, …, n) as follows:

$$R_{i}=PA-IT2FS-Z({\tilde{\tilde{A}}}_{1},{\tilde{\tilde{A}}}_{2},\ldots ,{\tilde{\tilde{A}}}_{m})=\left(\begin{array}{c} (\frac{\sum_{j=1}^{m}\omega_{j}(1+T({\tilde{\tilde{A}}}_{j}))a_{j1}^{U}}{\sum_{j=1}^{m}\omega_{j}(1+T({\tilde{\tilde{A}}}_{j}))},\frac{\sum_{j=1}^{m}\omega_{j}(1+T({\tilde{\tilde{A}}}_{j}))a_{j2}^{U}}{\sum_{j=1}^{m}\omega_{j}(1+T({\tilde{\tilde{A}}}_{j}))},\frac{\sum_{j=1}^{m}\omega_{j}(1+T({\tilde{\tilde{A}}}_{j}))a_{j3}^{U}}{\sum_{j=1}^{m}\omega_{j}(1+T({\tilde{\tilde{A}}}_{j}))},\frac{\sum_{j=1}^{m}\omega_{j}(1+T({\tilde{\tilde{A}}}_{j}))a_{j4}^{U}}{\sum_{j=1}^{m}\omega_{j}(1+T({\tilde{\tilde{A}}}_{j}))};);{min}_{j=1,2,..,m}h_{jA}^{U}\\ ,(\frac{\sum_{j=1}^{m}\omega_{j}(1+T({\tilde{\tilde{A}}}_{j}))a_{j1}^{L}}{\sum_{j=1}^{m}\omega_{j}(1+T({\tilde{\tilde{A}}}_{j}))},\frac{\sum_{j=1}^{m}\omega_{j}(1+T({\tilde{\tilde{A}}}_{j}))a_{j2}^{L}}{\sum_{j=1}^{m}\omega_{j}(1+T({\tilde{\tilde{A}}}_{j}))},\frac{\sum_{j=1}^{m}\omega_{j}(1+T({\tilde{\tilde{A}}}_{j}))a_{j3}^{L}}{\sum_{j=1}^{m}\omega_{j}(1+T({\tilde{\tilde{A}}}_{j}))},\frac{\sum_{j=1}^{m}\omega_{j}(1+T({\tilde{\tilde{A}}}_{j}))a_{j4}^{L}}{\sum_{j=1}^{m}\omega_{j}(1+T({\tilde{\tilde{A}}}_{j}))});{min}_{j=1,2,..,m}h_{jA}^{L} \end{array}\right)$$ (30)

Where *ω*_*j*_ stands for the expert E_j_’s weight.

### 3.3. The integration of IT2F and Z-number

The method developed by Kahraman et al. is used in this research for defuzzification since it takes fewer calculations than other methods [56].

Let IT2FS-Z

Z = $\left(\tilde{\tilde{A}},\tilde{\tilde{B}})=[\left(a_{1}^{U},a_{2}^{U},a_{3}^{U},a_{4}^{U};h_{A}^{U}),(a_{1}^{L},a_{2}^{L},a_{3}^{L},a_{4}^{L};h_{A}^{L}),(b_{1}^{U},b_{2}^{U},b_{3}^{U},b_{4}^{U};h_{b}^{U}),(b_{1}^{L},b_{2}^{L},b_{3}^{L},b_{4}^{L};h_{b}^{L}\right)\right]$ then defuzzification can be calculated as:

$${FPS}_{B}=\frac{\left[\frac{\left(b_{4}^{U}-b_{1}^{U})+(h_{b}^{U}\times b_{2}^{U}-b_{1}^{U})+(h_{b}^{U}\times b_{3}^{U}-b_{1}^{U}\right)}{4}+b_{1}^{U}]+[\frac{\left(b_{4}^{L}-b_{1}^{L})+(h_{b}^{L}\times b_{2}^{L}-b_{1}^{L})+(h_{b}^{L}\times b_{3}^{L}-b_{1}^{L}\right)}{4}+b_{1}^{L}\right]}{2}$$ (31)

Then *FPS*_*B*_ is multiplied in part A according to Eq 8 and definition 18:

$${\;\tilde{\tilde{z}}}^{\alpha }=(\tilde{\tilde{A}},\sqrt{{FPS}_{b}})\to {\tilde{\tilde{z}}}^{\prime }=(\sqrt{{FPS}_{b}}\times \tilde{\tilde{A}})\overset{Eq.8}{\to }\;{\tilde{\tilde{z}}}^{\prime }=((\sqrt{{FPS}_{b}}\times a_{1}^{U},{\sqrt{{FPS}_{b}}\times a}_{2}^{U},{\sqrt{{FPS}_{b}}\times a}_{3}^{U},\sqrt{{FPS}_{b}}\times a_{4}^{U};h_{A}^{U}),({\sqrt{{FPS}_{b}}\times a}_{1}^{L},\sqrt{{FPS}_{b}}\times a_{2}^{L},\sqrt{{FPS}_{b}}\times a_{3}^{L},{\sqrt{{FPS}_{b}}\times a}_{4}^{L};h_{A}^{L}))$$ (32)

Like Eq 31, the first part of the Z-number was also defuzzified and the FPS value for part A (*FPS*_*A*_) obtained. A simple example is provided considering four experts in S1 Appendix to clarify the above explanations.

### 3.4. Converting FPS into FFP

The expert-rated fuzzy probability score (FPS) must be converted to calculate the fuzzy failure probability (FFP). The conventional conversion method [68] is:

$$FFP=\left\{ \begin{array}{l} \frac{1}{10^{K}}\;FPS\neq 0\\ 0\;FPS=0 \end{array}\right.$$ (33)

$$K={\left[\frac{1-FPS}{FPS}\right]}^{1/3}\times 2.301$$ (34)

The Onisawa method only holds in some circumstances. In this study, a more effective calculation method is proposed (Eq 35), along with the conversion of FPS to FFP by DNV standards.


$$K=\left\{ \begin{array}{c} -0.721\;\ln\;FPS+2.839,\;0\leq FPS\leq 0.2\\ 4.523-3.287FPS,\;0.2\leq FPS\leq 0.8\\ {\left[(1-FPS)/FPS\right]}^{0.445}\times 3.705,\;0.8\leq FPS\leq 1 \end{array}\right.$$ (35)

### 3.5. Mapping bow-tie in BN

The probability of the BEs derived using the IT2FS-Z technique were taken into account as the probabilities of root nodes occurrences in order to quantify the model. The bow-tie model was mapped in BN using a technique developed by Khakzad et al. Because BN is used as a dynamic tool for updating the probability of barriers and consequences in the next steps [26]. Each construction step meticulously addresses uncertainty to enhance the robustness and reliability of the Bayesian network. This is achieved through rigorous data validation procedures, ensuring the transparency and traceability of information at every stage. Each network layer demonstrably contributes to its overall predictive accuracy by reducing uncertainty and maintaining transparency. GeNIe 3.0 academic software was used to create and assess the Bayesian model. The BEs, intermediate, and top events in the BT are the BN model’s root, middle, and top nodes, respectively.

### 3.6. Sensitivity analysis in BN (CBEs identification)

The primary objective of the sensitivity analysis in this study is to pinpoint the critical basic events (CBEs) within the bow-tie model. CBEs represent the basic events (BEs) that most influence triggering the top event (TE) in the bow-tie model. Identifying CBEs is crucial for prioritizing risk mitigation strategies by honing in on the most impactful factors. The sensitivity analysis leverages a Bayesian network (BN) framework to evaluate the individual BEs’ impact on the TE.

To uncover sensitive nodes (CBEs), the sensitivity analysis employs the best and worst-case scenarios for each node in the BN as input. By applying these scenarios to specific BEs, the evidence (success/failure) for each BE is updated within the BN, considering the established prior probabilities and interrelations with other BEs in the network.

During the sensitivity analysis, all nodes except the BE under scrutiny remain unchanged, enabling a focused assessment of each BE’s influence on the TE’s probability. By monitoring the TE’s probability fluctuations following the evidence updates for each BE, we can identify the BEs causing the most significant changes. These identified BEs are designated as the critical basic events (CBEs). The sensitivity analysis yields valuable insights into the relative importance of BEs in shaping the overall risk profile of the system depicted by the bow-tie model.

### 3.7. Probability adapting

One of the most significant and beneficial uses of Bayesian networks is sequence learning, which can also be thought of as probability adaptation [69]. Due to this feature, the posterior probability of consequences was calculated taking into account the posterior probability of CBEs and their occurrences over the past five years.

The prior probability of the BEs in the bow-tie diagram, including the fault tree and the event tree, is known. These prior probabilities are assumed to follow the beta distribution and were obtained based on the technical knowledge of experts. Then, five years of data, including incidents, near misses, and accident reports, were analyzed to determine the frequency of success and failure of the primary safety barrier (BEs). According to this information, the posterior probability of CBEs is calculated using Eq 25. This application updates probabilities based on new data received over time.

The updated probability of the CBEs enters the Bayesian network and replaces the prior probability of these events. Following this stage, the probability of failure of barriers (RPB, DPB, IPB, EPB, HFB, M&OB) and the probability of consequences, including near miss, mishap, incident, and accident, are updated.

This operation was calculated in IT2FS and IT2FS-Z modes and with different variances (10^−4^ and 10^−5^) to enable comparison of the results.

## 4. A case study of gas release from spherical tank

The method explained in Section 3 is utilized to conduct a dynamic risk evaluation of a spherical tank. This study was conducted in a refinery located in the northwest region of Iran in 2023. The refinery consists of four spherical tanks, each with 2,337 *m*^3^ of raw materials for producing LPG, and one spherical tank with 635 *m*^3^ of LPG.

### 4.1. Bow-tie

**Fig 5:**
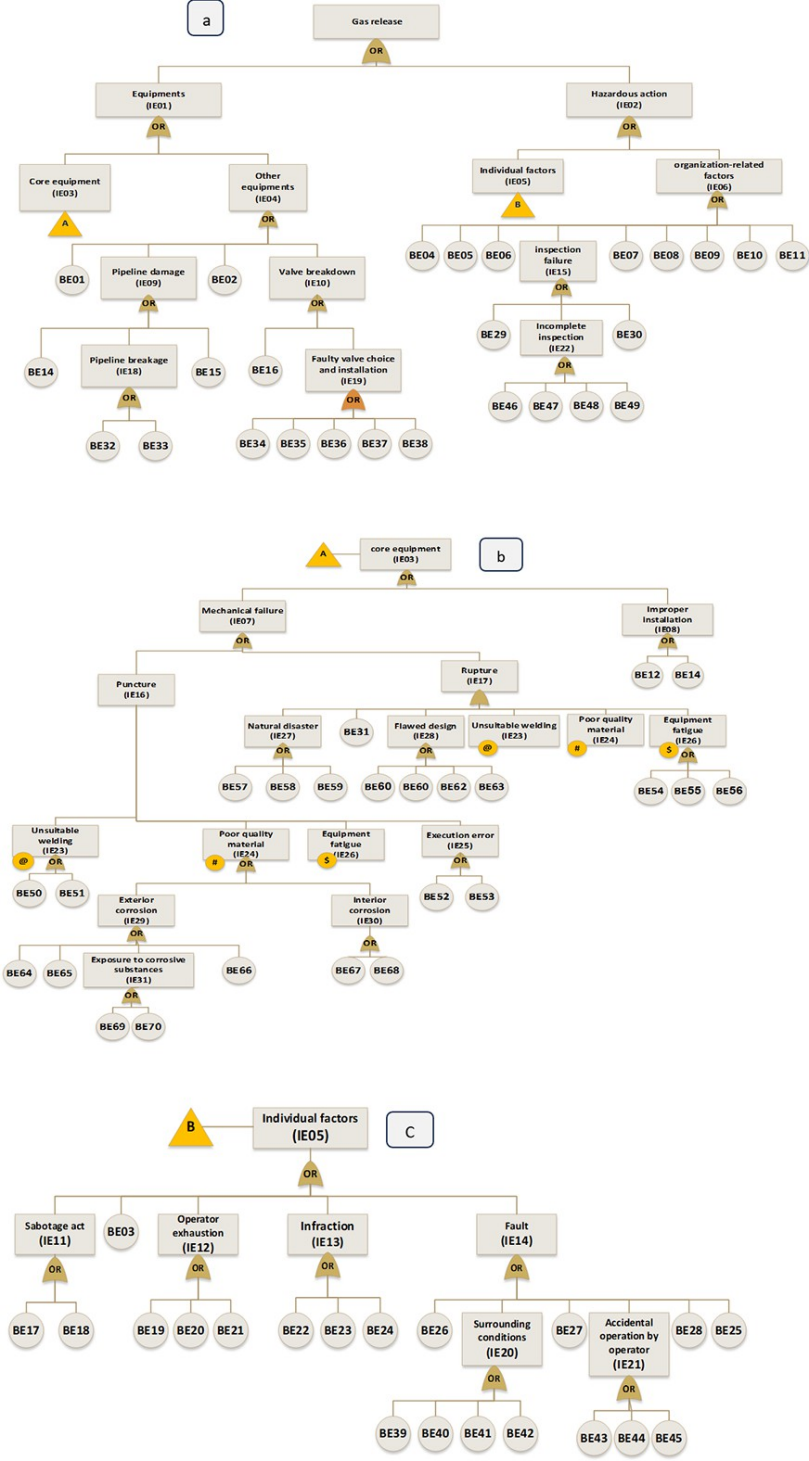
Fault tree of gas release in spherical tank.

Gas release from the spherical tank is considered a Top Event (TE) to draw the fault tree. Intermediate events (IE)and basic events (BE) that caused the gas release are illustrated as a part of a fault tree in Fig 5. Table 4 contains descriptions of the 70 basic events that were identified.

**Table 4:** The basic events of fault tree and their symbols.

Symbol	Event description	Symbol	Event description
**BE01**	Leakage from pressure device	**BE36**	Pressure safety valve failure
**BE02**	Pump failure	**BE37**	The valve dimensions do not meet the required standards
**BE03**	Smoking in prohibited areas during loading	**BE38**	limited valve accessibility
**BE04**	Inability to learn from past accidents/incidents	**BE39**	work environment unsuitable for humans
**BE05**	Weak safety culture	**BE40**	Inappropriate tools
**BE06**	Not providing essential education or training	**BE41**	Disturbing noise and lighting
**BE07**	Poor permit issuance system	**BE42**	Excessive heat/cold
**BE08**	Unsatisfactory maintenance and repair	**BE43**	Unintentionally opened valve
**BE09**	Limited information access	**BE44**	Mishandled license
**BE10**	Poor management liability	**BE45**	Unplanned displacement of a tanker during loading
**BE11**	Weak discipline and organization	**BE46**	Delayed detection of covert hazards
**BE12**	Foundation subsidence	**BE47**	Insufficient supervision in emergencies
**BE13**	Inappropriate arrangement	**BE48**	Failing to recognize risky conduct
**BE14**	Seal breakdown in pipes	**BE49**	Perfunctory inspections
**BE15**	Weld fracture	**BE50**	Defective weld seam design
**BE16**	Stuck LPG valve due to freezing	**BE51**	Structural deficiency in the weld seam
**BE17**	Terrorist incident	**BE52**	Damage to machinery
**BE18**	Retaliatory operation	**BE53**	Improper placement
**BE19**	Operator’s insufficient physical fitness for work conditions	**BE54**	Localized membrane stress at the nozzle connection
**BE20**	Limited rest caused by working non-standard shifts	**BE55**	Stress (Shear stress, Tensile stress, Compressive stress)
**BE21**	Ineligible health status of the operator	**BE56**	Vibration
**BE22**	Skipping checklist items	**BE57**	Deluge
**BE23**	Disregarding leak detection system alerts	**BE58**	Earthquake
**BE24**	Disregard tank operation protocols	**BE59**	Ground subsidence or settlement
**BE25**	Misestimating tank pressure levels from gauges	**BE60**	Inner structure
**BE26**	Permitting work without proper assessment	**BE61**	Substandard strength
**BE27**	Improper reaction to multiple alarms	**BE62**	The short distance between the tanks
**BE28**	Inappropriate decision-making during the operation	**BE63**	Fault in the design of the discharge environment
**BE29**	Lack of inspection	**BE64**	Coating degradation
**BE30**	Ineffective operating procedures	**BE65**	Excessive heat
**BE31**	The explosion of an adjacent unit/facility	**BE66**	Inadequate cathodic protection implementation
**BE32**	Process variations upstream	**BE67**	H2S
**BE33**	Vehicle crash	**BE68**	WATER
**BE34**	The valves are installed too closely	**BE69**	Hydrocarbon corrosive gases around the spherical tank
**BE35**	Valve control system malfunction	**BE70**	Humidity

**Fig 6:**
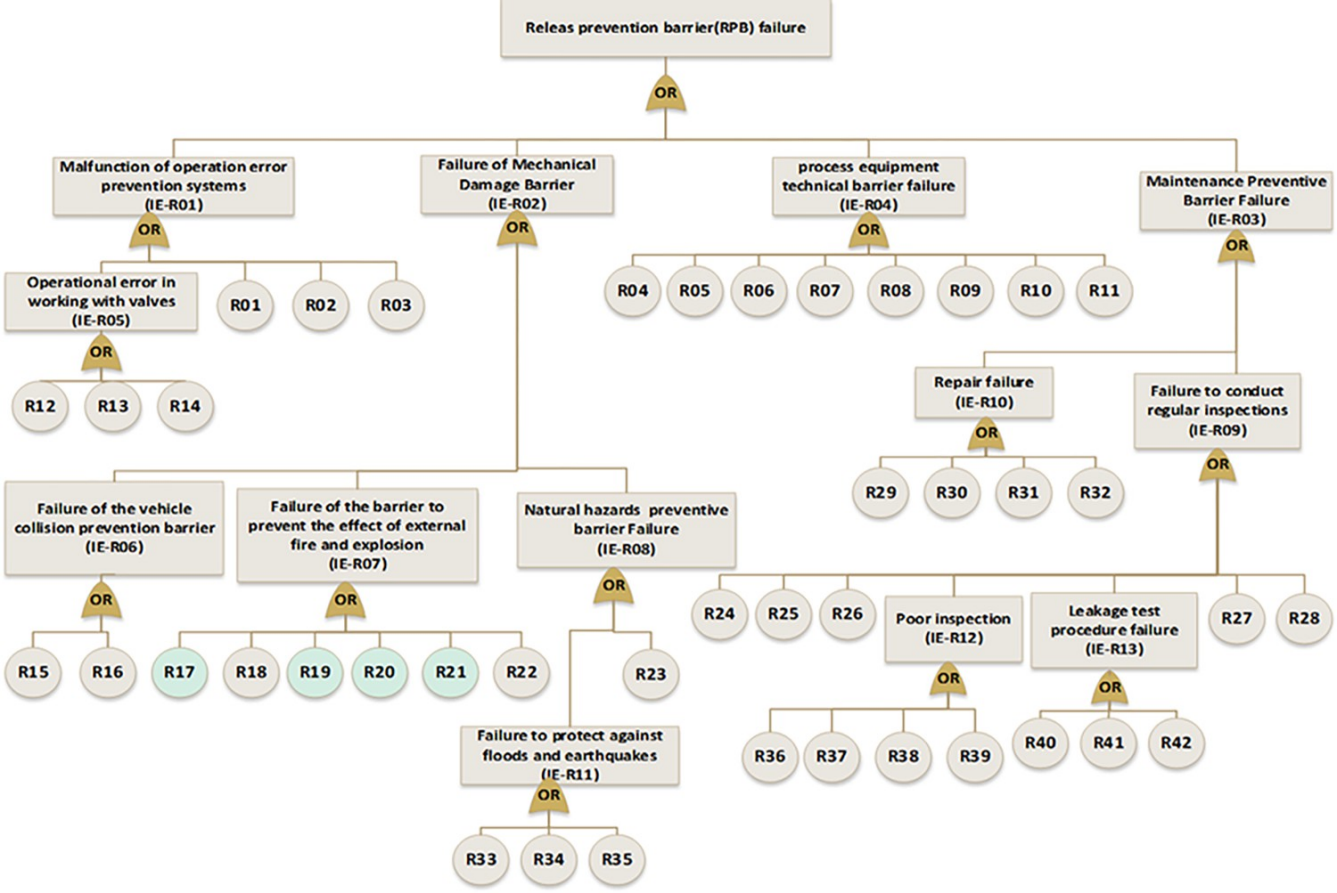
Fault tree of release prevention barrier (RPB) failure.

**Fig 7:**
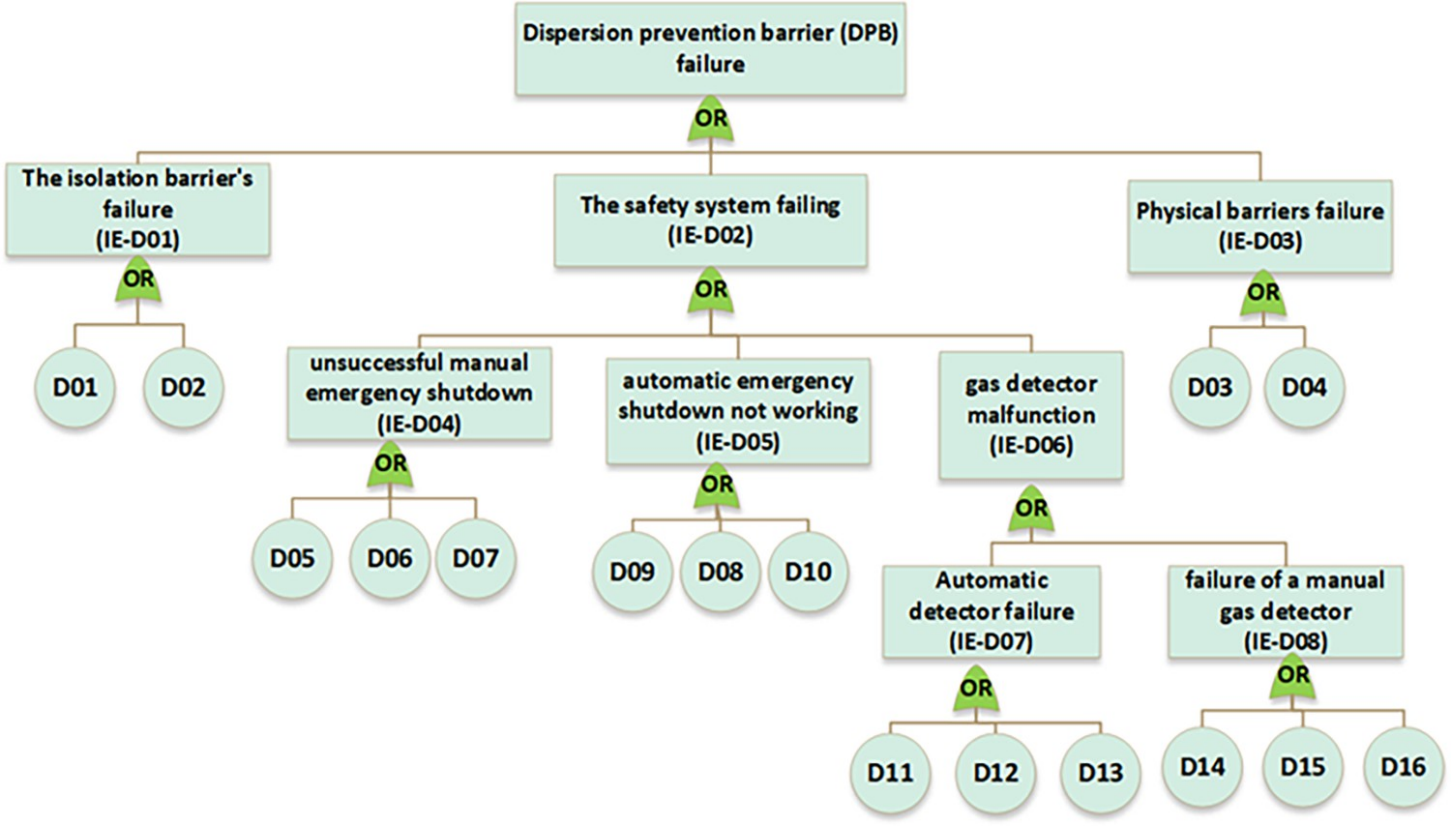
Fault tree of dispersion prevention barrier (DPB) failure.

**Fig 8:**
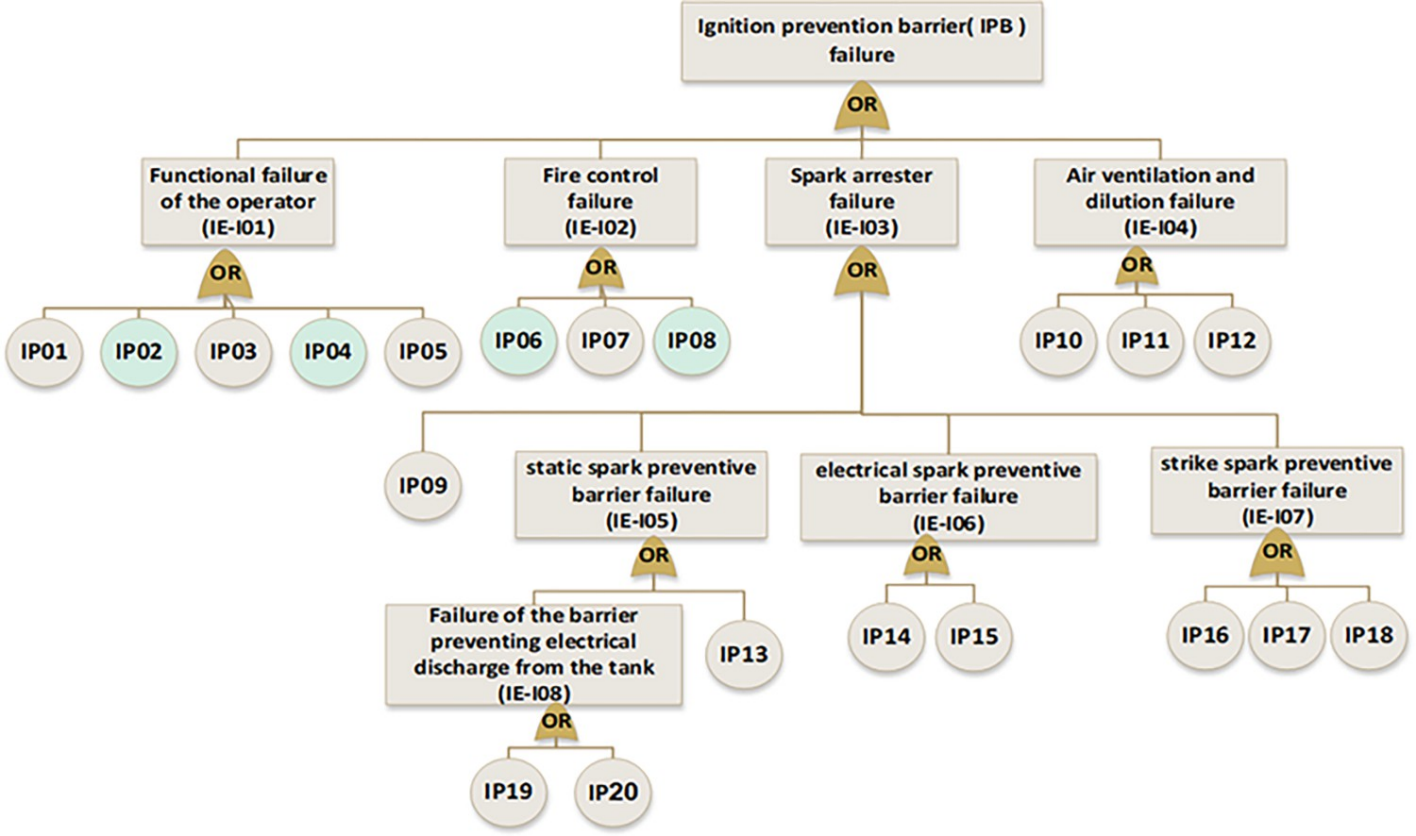
Fault tree of ignition prevention barrier (IPB) failure.

**Fig 9:**
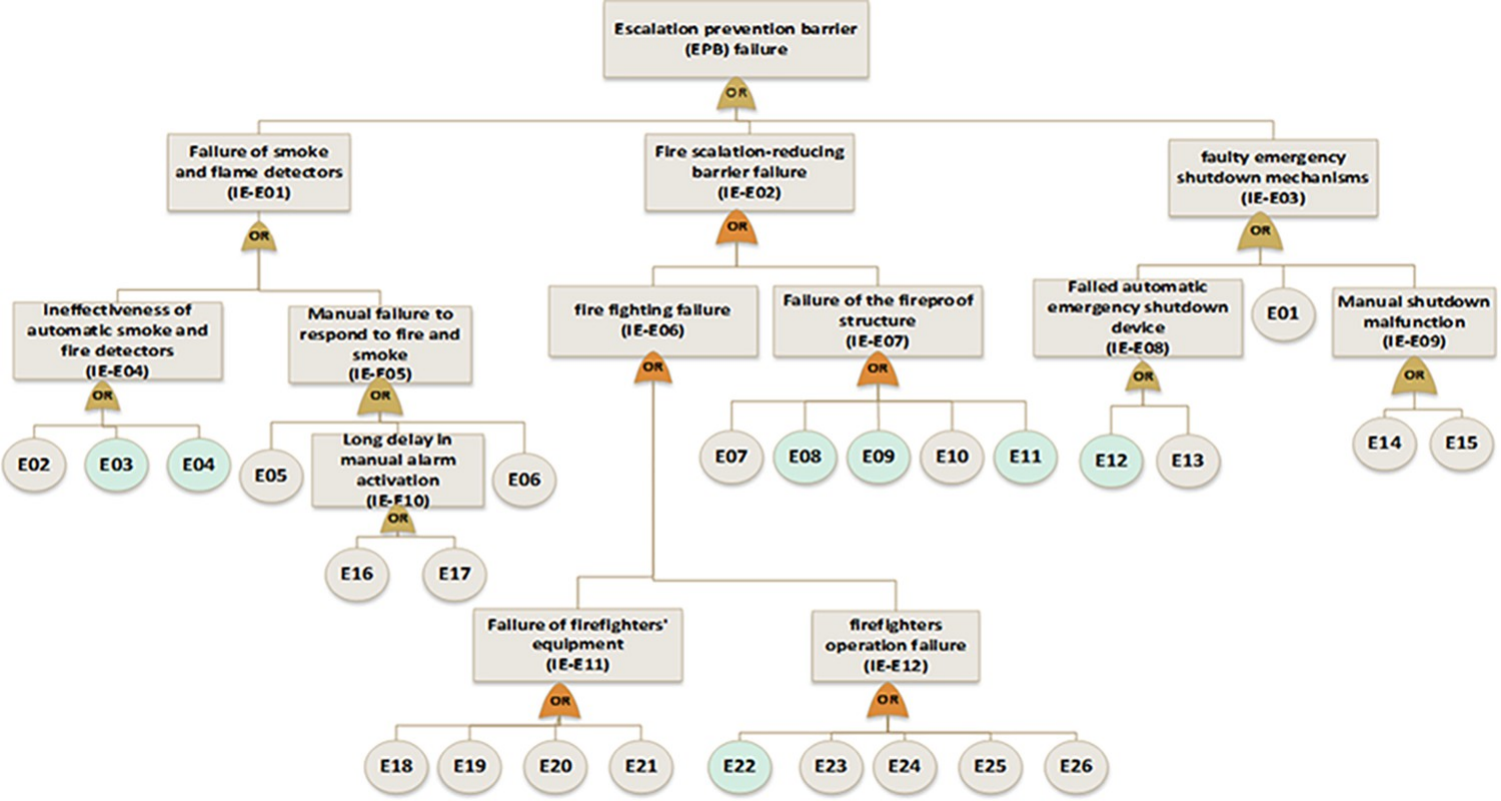
Fault tree of escalation prevention barrier (EPB) failure.

**Fig 10:**
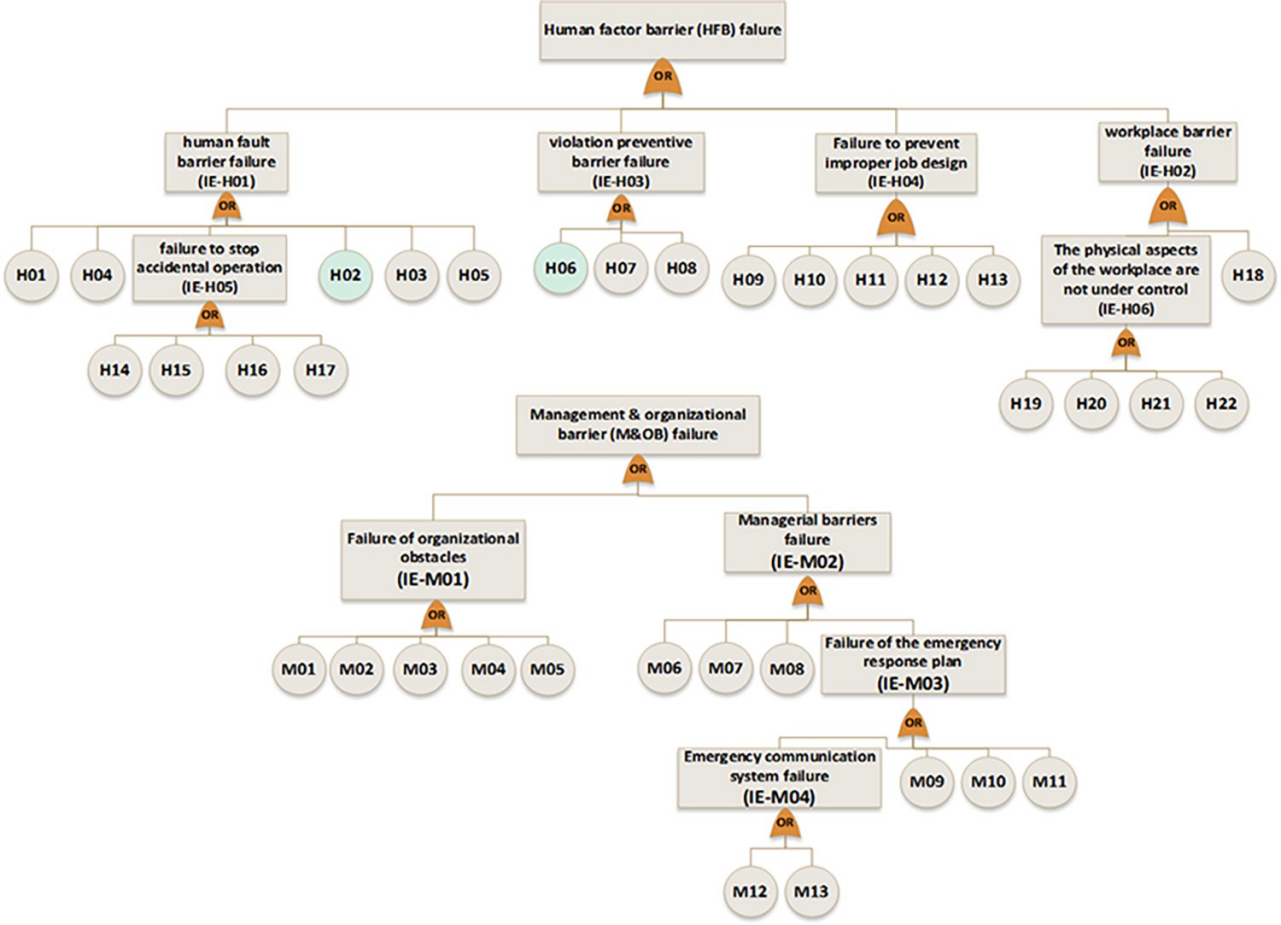
Fault tree of human factor barrier (HFB) failure and management &organizational barrier(M&OB) failure.

In the next step, near miss, mishap, incident, and accident were considered consequences of gas release. According to the SHIPP concept (Fig 4), a fault tree was created to assess the failure probability of each barrier. The release prevention barrier (RPB) fault tree comprises 42 BEs (symbolized as R), the extended dispersion prevention barrier (DPB) fault tree comprises 16 BEs (symbolized as D), the ignition prevention barrier (IPB) fault tree encompasses 20 BEs (symbolized as IP), the escalation prevention barrier (EPB) fault tree contains 26 BEs (symbolized as E). Additionally, there are 22 BEs associated with the human factor barrier (HFB) fault tree (symbolized as H), and the fault tree for management and organization barrier (M&OB) incorporates 13 BEs (symbolized as M), as illustrated in Figs 6 to 10. Detailed descriptions of these basic events are provided in Table 5.

**Table 5:** The symbols and descriptions of basic event of barriers’ failure fault tree.

Symbol	Event description	Symbol	Event description
**R01**	Disregard of work permit	**IP13**	Failure of the barrier to prevent electrical discharge from the body
**R02**	Inadequate performance of active safety measures	**IP14**	Unauthorized mobile device use
**R03**	Defect identification failure	**IP15**	Welding without a permit
**R04**	system failure	**IP16**	Failure to replace worn valves (probability of sparks)
**R05**	Liquid level indicator failure	**IP17**	A spark caused by the collision of equipment
**R06**	Malfunctioning pressure gauge	**IP18**	Excessive use of equipment
**R07**	Inner wall corrosion prevention barrier failure	**IP19**	Accumulation of electricity
**R08**	Faulty safety valve	**IP20**	Faulty grounding system
**R09**	Failure of external corrosion prevention barrier	**E01**	Faulty sealing of the leak
**R10**	aging prevention barrier failure	**E02**	Using detectors incompatible with the type of fire
**R11**	Valve seal deflagration	**E03**	Insufficient detector coverage
**R12**	Error in setting the manual valves to the required level	**E04**	Defective detector
**R13**	Lack of identification labels on valves and equipment	**E05**	Lack of training on proper handling of fire or smoke
**R14**	Automatic valve position detection error	**E06**	Failure of the operator to visually detect the fire
**R15**	Failure to create protection or fence for tanks	**E07**	Delayed emptying of spherical tanks
**R16**	Failure to determine the safety margin for pipes	**E08**	Cooling system failure
**R17**	The poor reaction of firefighters	**E09**	Failure of spherical tanks’ body insulation
**R18**	Delay in firefighting operations	**E10**	Lack of standard distance between tanks and other facilities
**R19**	Cooling system failure	**E11**	Lack of standard distance between tanks
**R20**	Lack of standard distance between tanks	**E12**	A sensor connected to the emergency shutdown system failed
**R21**	Loss of spherical tanks’ body insulation	**E13**	Emergency shut-off valve failure
**R22**	The extinguishing system failure (e.g., water pipe blockage, low foam pressure, etc.)	**E14**	Operator warning failure
**R23**	Lightning rod failure	**E15**	Failure to enable manual emergency shutdown
**R24**	Internal corrosion inspection failure	**E16**	Defective manual alarm switch
**R25**	Failure of external corrosion inspection	**E17**	Failure to place the manual switch in the proper view
**R26**	Delay in reporting results	**E18**	Low-capacity fire suppression apparatus
**R27**	Failure to inspect the tank base and foundation	**E19**	Damage to fire water pipes
**R28**	Welding inspection failure	**E20**	Water freezing in the pipe
**R29**	Use of low-quality materials	**E21**	Worn and old fire engine
**R30**	Insufficient experience of repairmen	**E22**	The poor reaction of firefighters
**R31**	Failure to test equipment after repair and installation	**E23**	Lack of sufficient fire drills and training throughout the year
**R32**	Installing the wrong equipment or the wrong standard	**E24**	Firefighters fatigue
**R33**	Failure to properly implement the 5S program around the tank	**E25**	Lack of coordination of different firefighting teams (lack of proper management)
**R34**	Weak tank foundation	**E26**	Firemen wearing inadequate personal protective equipment
**R35**	Hazardous nearby structures to the tank	**H01**	Operator’s Physical limitation
**R36**	Test and measurement equipment error	**H02**	Inadequate supervision or lack of supervision
**R37**	Insufficient experience of inspectors	**H03**	Lack of motivation to enhance skills
**R38**	Inadequate knowledge of the visiting operator	**H04**	Erroneous decision-making during operation
**R39**	Lack of comprehensive and clear inspection instructions	**H05**	Lack of relevant knowledge and skills
**R40**	Failure to identify small leaks	**H06**	Inadequate supervision or lack of supervision
**R41**	A minor leak search program is not followed	**H07**	Complying with security instructions failure
**R42**	Failure to test leakage control devices	**H08**	Ignoring the job status of employees
**D01**	A valve to stop the incoming flow is either absent or malfunctioning	**H09**	Stressful work process
**D02**	Long wait time for operator responses	**H10**	Irregular work shift
**D03**	Physical barriers not present at the entrance to enclosed spaces and Underground floors	**H11**	Excessive night work
**D04**	using imperfect physical barriers	**H12**	Improper placement of equipment or poor partitioning of the work environment
**D05**	Emergency shut-off valve’s resemblance to other nearby valves	**H13**	Ambiguous job task definition
**D06**	Lack of easy access to the emergency stop valve	**H14**	Failure to check the conditions before issuing a work permit
**D07**	Lack of a manual shut-off valve	**H15**	Failure of equipment that prevents incorrect action
**D08**	Late activation of valve	**H16**	Lack of identification labels on valves and equipment
**D09**	Malfunction of the emergency stop controller	**H17**	Inappropriate tool
**D10**	The sensor connected to the emergency shutdown system failed	**H18**	Failure to control undesirable chemical factors in the workplace (gas smell, fumes, vapors, etc.)
**D11**	Insufficient detector coverage	**H19**	Lighting maintenance failure
**D12**	Defective gas monitoring device	**H20**	Volume control failure
**D13**	Sensor operation only at a very high gas concentration	**H21**	Vibration control failure
**D14**	poor quality manual gas detectors	**H22**	Unfavorable temperature
**D15**	Lack of operator familiarity with manual equipment operation	**M01**	Insufficient safety planning
**D16**	inadequate manual equipment sensitivity for low concentration	**M02**	Poor maintenance system
**IP01**	using the wrong permit to work	**M03**	Improper control system
**IP02**	Inadequate supervision or lack of supervision	**M04**	Poor organization
**IP03**	Ignoring the no smoking sign	**M05**	Weak inspection system
**IP04**	Failure to check the conditions before issuing a work permit	**M06**	Low accountability
**IP05**	Doing hot work without a permit	**M07**	Ignoring standards and audits
**IP06**	Defective detector	**M08**	Poor management decision
**IP07**	There is no detector	**M09**	Lack of support team for major events
**IP08**	Insufficient detector coverage	**M10**	Lack of emergency command instructions
**IP09**	lightning arrester system failure	**M11**	lack of specific training programs for firefighters and emergency response team
**IP10**	Failure to dilute with inert gas or water spray	**M12**	Off-site communication failure for emergencies
**IP11**	Lack of ventilation equipment	**M13**	On-site communication failure for emergencies
**IP12**	The ventilation system works but is ineffective	**-**	

The IT2FS-Z and IT2FS methods were utilized to acquire the prior likelihood of BEs. The FFP, K, and FPS values obtained for the fault tree’s BEs associated with the TE are displayed in Tables 6 and 7, respectively. Additionally, Tables 8 and 9 show the prior probability of the BEs linked to the failure of RPB, DPB, IPB, and EPB barriers.

**Table 6:** FPS and FFP values of the basic events of the top event fault tree based on IT2FS-Z.

	FPS	K	FFP		FPS	K	FFP
**BE01**	0.280276416	3.601731422	0.000250189	**BE36**	0.4920903	2.905499184	0.001243085
**BE02**	0.513047102	2.836614175	0.001456753	**BE37**	0.234645262	3.751721025	0.000177125
**BE03**	0.337173217	3.414711637	0.000384847	**BE38**	0.338685694	3.409740125	0.000389278
**BE04**	0.441856584	3.070617408	0.000849929	**BE39**	0.38472116	3.258421548	0.000551542
**BE05**	0.445160126	3.059758666	0.000871448	**BE40**	0.43857163	3.081415053	0.000829058
**BE06**	0.358319458	3.34520394	0.000451644	**BE41**	0.359963776	3.339799069	0.0004573
**BE07**	0.379283239	3.276295995	0.000529303	**BE42**	0.382229695	3.266610992	0.000541239
**BE08**	0.302607999	3.528327507	0.00029626	**BE43**	0.49386722	2.899658448	0.001259916
**BE09**	0.281214462	3.598648062	0.000251972	**BE44**	0.337957703	3.41213303	0.000387139
**BE10**	0.303465798	3.525507922	0.000298189	**BE45**	0.439764329	3.077494649	0.000836576
**BE11**	0.379379773	3.275978687	0.000529689	**BE46**	0.374159287	3.293138425	0.000509169
**BE12**	0.151245549	4.20086129	6.29707E-05	**BE47**	0.442557302	3.068314148	0.000854448
**BE13**	0.350507707	3.370881168	0.000425715	**BE48**	0.299334832	3.539086406	0.00028901
**BE14**	0.48804477	2.918796842	0.0012056	**BE49**	0.298385642	3.542206394	0.000286942
**BE15**	0.512290323	2.83910171	0.001448433	**BE50**	0.29205209	3.563024779	0.000273511
**BE16**	0.505533387	2.861311758	0.001376221	**BE51**	0.383615409	3.262056152	0.000546945
**BE17**	0.167467663	4.127401767	7.45759E-05	**BE52**	0.28374459	3.590331534	0.000256843
**BE18**	0.261587504	3.663161874	0.000217189	**BE53**	0.51467953	2.831248386	0.001474863
**BE19**	0.337641637	3.413171938	0.000386214	**BE54**	0.395599341	3.222664968	0.000598873
**BE20**	0.353926711	3.359642901	0.000436875	**BE55**	0.423931869	3.129535945	0.000742103
**BE21**	0.353910926	3.359694786	0.000436823	**BE56**	0.283943309	3.589678345	0.00025723
**BE22**	0.268000527	3.642082266	0.000227991	**BE57**	0.30012904	3.536475846	0.000290753
**BE23**	0.321447969	3.466400527	0.000341664	**BE58**	0.200696271	3.863311357	0.00013699
**BE24**	0.378684095	3.278265378	0.000526908	**BE59**	0.284463074	3.587969876	0.000258244
**BE25**	0.215339025	3.815180624	0.000153045	**BE60**	0.295402969	3.552010442	0.000280537
**BE26**	0.403787869	3.195749273	0.000637163	**BE61**	0.477186255	2.95448878	0.001110481
**BE27**	0.268648125	3.639953612	0.000229111	**BE62**	0.372965564	3.29706219	0.000504589
**BE28**	0.234003309	3.753831125	0.000176266	**BE63**	0.24317131	3.723695903	0.000188931
**BE29**	0.366299507	3.318973521	0.000479763	**BE64**	0.187955644	4.04418703	9.0326E-05
**BE30**	0.374033457	3.293552026	0.000508684	**BE65**	0.385847075	3.254720665	0.000556262
**BE31**	0.328627489	3.442801445	0.000360744	**BE66**	0.303348789	3.525892531	0.000297925
**BE32**	0.446826096	3.054282623	0.000882505	**BE67**	0.298710443	3.541138772	0.000287648
**BE33**	0.170554989	4.114230912	7.68722E-05	**BE68**	0.434849763	3.093648829	0.00080603
**BE34**	0.309209284	3.506629082	0.000311438	**BE69**	0.328978345	3.441648181	0.000361703
**BE35**	0.479254571	2.947690225	0.001128002	**BE70**	0.385023083	3.257429126	0.000552804

**Table 7:** FPS and FFP values of the basic events of the top event fault tree based on IT2FS.

	FPS	K	FFP		FPS	K	FFP
**BE01**	0.44591094	3.057290739	0.000876414	**BE36**	0.590266267	2.58279478	0.002613396
**BE02**	0.648834536	2.39028088	0.004071169	**BE37**	0.35843219	3.34483339	0.000452029
**BE03**	0.468669404	2.982483669	0.001041157	**BE38**	0.462316033	3.0033672	0.000992277
**BE04**	0.594961022	2.567363121	0.002707927	**BE39**	0.481547937	2.940151932	0.001147752
**BE05**	0.581924941	2.610212717	0.002453507	**BE40**	0.580010259	2.616506278	0.002418208
**BE06**	0.549993605	2.715171019	0.001926766	**BE41**	0.512831374	2.837323275	0.001454376
**BE07**	0.521597259	2.808509809	0.00155414	**BE42**	0.503300244	2.868652099	0.001353156
**BE08**	0.498273631	2.885174576	0.001302643	**BE43**	0.635931723	2.432692426	0.00369239
**BE09**	0.397181936	3.217462975	0.00060609	**BE44**	0.435087582	3.092867119	0.000807482
**BE10**	0.47897748	2.948601023	0.001125639	**BE45**	0.570364376	2.648212295	0.002247955
**BE11**	0.547614941	2.722989689	0.001892389	**BE46**	0.484270254	2.931203677	0.001171646
**BE12**	0.220322258	3.798800736	0.000158928	**BE47**	0.588028825	2.590149253	0.002569513
**BE13**	0.493171692	2.901944648	0.001253301	**BE48**	0.40229972	3.20064082	0.000630027
**BE14**	0.663582938	2.341802884	0.004551946	**BE49**	0.430505912	3.107927067	0.000779961
**BE15**	0.681165809	2.284007986	0.005199864	**BE50**	0.418152111	3.148534012	0.00071034
**BE16**	0.687141978	2.264364318	0.005440461	**BE51**	0.486365201	2.924317583	0.001190371
**BE17**	0.203702492	3.853429909	0.000140143	**BE52**	0.399322665	3.210426402	0.00061599
**BE18**	0.357111657	3.349173982	0.000447534	**BE53**	0.65037141	2.385229176	0.004118801
**BE19**	0.451040853	3.040428715	0.000911111	**BE54**	0.533353186	2.769868076	0.00169876
**BE20**	0.535613785	2.762437488	0.001728075	**BE55**	0.595822811	2.56453042	0.002725647
**BE21**	0.458647035	3.015427196	0.000965101	**BE56**	0.3684355	3.311952511	0.000487582
**BE22**	0.365983367	3.320012672	0.000478616	**BE57**	0.370911887	3.303812629	0.000496807
**BE23**	0.445614189	3.058266159	0.000874448	**BE58**	0.273235615	3.624874534	0.000237206
**BE24**	0.495701099	2.893630487	0.001277525	**BE59**	0.3877312	3.248527545	0.000564251
**BE25**	0.349183778	3.375232922	0.00042147	**BE60**	0.387421522	3.249545456	0.00056293
**BE26**	0.535525546	2.762727529	0.001726921	**BE61**	0.66751628	2.328873988	0.004689494
**BE27**	0.365699151	3.320946891	0.000477588	**BE62**	0.533646079	2.768905339	0.00170253
**BE28**	0.320611344	3.469150512	0.000339508	**BE63**	0.29539556	3.552034793	0.000280521
**BE29**	0.463183262	3.000516619	0.000998811	**BE64**	0.230559974	3.765149367	0.000171732
**BE30**	0.494430578	2.897806689	0.001265299	**BE65**	0.488993371	2.915678788	0.001214287
**BE31**	0.451871474	3.037698464	0.000916857	**BE66**	0.393601959	3.229230362	0.000589888
**BE32**	0.595689256	2.564969414	0.002722893	**BE67**	0.428915302	3.113155401	0.000770628
**BE33**	0.258448429	3.673480012	0.00021209	**BE68**	0.601579805	2.545607181	0.002847035
**BE34**	0.397887414	3.215144071	0.000609335	**BE69**	0.43888162	3.080396117	0.000831005
**BE35**	0.607525931	2.526062265	0.002978089	**BE70**	0.499222522	2.882055569	0.001312032

**Table 8:** FPS and FFP values of the basic events in the barrier failure fault tree based on IT2FS-Z.

	FPS	K	FFP		FPS	K	FFP
**R01**	0.468046832	2.984530063	0.001036263	**IP13**	0.505758327	2.86057238	0.001378566
**R02**	0.473065421	2.968033962	0.001076381	**IP14**	0.397055271	3.217879323	0.000605509
**R03**	0.479211003	2.947833433	0.00112763	**IP15**	0.220454888	3.798364783	0.000159087
**R04**	0.461481573	3.006110069	0.00098603	**IP16**	0.526912037	2.791040133	0.001617931
**R05**	0.436211397	3.089173137	0.00081438	**IP17**	0.503991663	2.866379403	0.001360256
**R06**	0.500986806	2.876256369	0.001329669	**IP18**	0.44574852	3.057824615	0.000875337
**R07**	0.487931564	2.919168948	0.001204567	**IP19**	0.516562777	2.825058153	0.001496035
**R08**	0.468694514	2.982401133	0.001041355	**IP20**	0.461343594	3.006563607	0.000985
**R09**	0.472643841	2.969419696	0.001072952	**E01**	0.42377299	3.130058181	0.000741211
**R10**	0.476379114	2.957141852	0.001103718	**E02**	0.200964506	3.86242967	0.000137268
**R11**	0.454742605	3.028261059	0.000936999	**E03**	0.434694116	3.094160442	0.000805081
**R12**	0.486063639	2.925308819	0.001187657	**E04**	0.439161019	3.07947773	0.000832765
**R13**	0.461834964	3.004948475	0.00098867	**E05**	0.273820985	3.622950422	0.000238259
**R14**	0.44331154	3.065834968	0.00085934	**E06**	0.446250455	3.056174755	0.000878669
**R15**	0.392434461	3.233067927	0.000584699	**E07**	0.461813214	3.005019964	0.000988508
**R16**	0.32355348	3.459479711	0.000347152	**E08**	0.479958818	2.945375365	0.00113403
**R17**	0.323397235	3.459993287	0.000346742	**E09**	0.405756527	3.189278297	0.000646728
**R18**	0.416339183	3.154493107	0.000700659	**E10**	0.402561477	3.199780427	0.000631276
**R19**	0.479958818	2.945375365	0.00113403	**E11**	0.515221239	2.829467789	0.001480922
**R20**	0.515221239	2.829467789	0.001480922	**E12**	0.483818724	2.932687853	0.001167649
**R21**	0.405756527	3.189278297	0.000646728	**E13**	0.441782185	3.070861959	0.00084945
**R22**	0.387460853	3.249416175	0.000563098	**E14**	0.473359849	2.967066176	0.001078782
**R23**	0.320012721	3.471118186	0.000337973	**E15**	0.472029273	2.971439779	0.001067973
**R24**	0.470932921	2.975043488	0.001059148	**E16**	0.333527002	3.426696743	0.000374372
**R25**	0.305832014	3.517730169	0.000303578	**E17**	0.286819447	3.580224478	0.000262891
**R26**	0.42381539	3.129918812	0.000741449	**E18**	0.476485335	2.956792704	0.001104606
**R27**	0.342860964	3.396016012	0.000401776	**E19**	0.33408519	3.424861979	0.000375957
**R28**	0.392550512	3.232686468	0.000585212	**E20**	0.38607756	3.25396306	0.000557233
**R29**	0.386344251	3.253086448	0.000558359	**E21**	0.424111306	3.128946136	0.000743111
**R30**	0.308913442	3.507601517	0.000310741	**E22**	0.323397235	3.459993287	0.000346742
**R31**	0.330545517	3.436496886	0.000366019	**E23**	0.340172163	3.404854099	0.000393682
**R32**	0.205214394	3.848460287	0.000141755	**E24**	0.447726806	3.051321989	0.000888542
**R33**	0.359556564	3.341137574	0.000455892	**E25**	0.418081275	3.148766848	0.000709959
**R34**	0.145621699	4.228181792	5.91314E-05	**E26**	0.354553172	3.357583722	0.000438951
**R35**	0.429515069	3.111183968	0.000774134	**H01**	0.209021301	3.835946985	0.000145899
**R36**	0.371109025	3.303164634	0.000497548	**H02**	0.345560768	3.387141755	0.00041007
**R37**	0.282656835	3.593906983	0.000254738	**H03**	0.424377242	3.128072006	0.000744609
**R38**	0.388001977	3.247637502	0.000565409	**H04**	0.347791367	3.379809776	0.000417052
**R39**	0.312920414	3.4944306	0.000320309	**H05**	0.279416833	3.604556871	0.000248567
**R40**	0.545832431	2.728848798	0.00186703	**H06**	0.345560768	3.387141755	0.00041007
**R41**	0.434500321	3.094797445	0.000803901	**H07**	0.41834258	3.147907941	0.000711364
**R42**	0.32327957	3.460380052	0.000346434	**H08**	0.445855761	3.057472113	0.000876048
**D01**	0.370488632	3.305203867	0.000495218	**H09**	0.461807209	3.005039705	0.000988463
**D02**	0.382619906	3.265328369	0.00054284	**H10**	0.325826739	3.452007508	0.000353177
**D03**	0.506911289	2.856782593	0.001390649	**H11**	0.343741403	3.393122007	0.000404462
**D04**	0.432727073	3.10062611	0.000793184	**H12**	0.36257702	3.331209337	0.000466434
**D05**	0.372764618	3.297722699	0.000503822	**H13**	0.365204536	3.32257269	0.000475803
**D06**	0.342956568	3.39570176	0.000402067	**H14**	0.356230095	3.352071679	0.000444558
**D07**	0.227827889	3.774129729	0.000168217	**H15**	0.332383149	3.430456589	0.000371145
**D08**	0.385201387	3.256843043	0.00055355	**H16**	0.461834964	3.004948475	0.00098867
**D09**	0.396872888	3.218478819	0.000604674	**H17**	0.433007869	3.099703133	0.000794871
**D10**	0.483818724	2.932687853	0.001167649	**H18**	0.309647955	3.505187173	0.000312473
**D11**	0.434694116	3.094160442	0.000805081	**H19**	0.465695876	2.992257655	0.001017987
**D12**	0.385307878	3.256493004	0.000553996	**H20**	0.329032324	3.44147075	0.000361851
**D13**	0.432955512	3.099875231	0.000794556	**H21**	0.34956873	3.373967584	0.0004227
**D14**	0.498861973	2.883240696	0.001308457	**H22**	0.328496131	3.443233216	0.000360385
**D15**	0.322343505	3.463456898	0.000343988	**M01**	0.350147309	3.372065796	0.000424555
**D16**	0.458784305	3.014975989	0.000966104	**M02**	0.339477007	3.407139077	0.000391616
**IP01**	0.218283561	3.805501935	0.000156494	**M03**	0.430377152	3.108350303	0.000779201
**IP02**	0.345560768	3.387141755	0.00041007	**M04**	0.281621087	3.597311488	0.000252748
**IP03**	0.241719574	3.728467759	0.000186867	**M05**	0.422737155	3.133462972	0.000735423
**IP04**	0.356230095	3.352071679	0.000444558	**M06**	0.305336955	3.519357429	0.000302442
**IP05**	0.164155242	4.141805685	7.2143E-05	**M07**	0.32454079	3.456234425	0.000349756
**IP06**	0.439161019	3.07947773	0.000832765	**M08**	0.442582182	3.068232367	0.000854609
**IP07**	0.322990879	3.461328979	0.000345677	**M09**	0.36342457	3.328423437	0.000469436
**IP08**	0.434694116	3.094160442	0.000805081	**M10**	0.374826917	3.290943925	0.000511748
**IP09**	0.312666536	3.495265096	0.000319694	**M11**	0.288039419	3.576214428	0.00026533
**IP10**	0.427751033	3.116982354	0.000763867	**M12**	0.449331121	3.046048605	0.000899397
**IP11**	0.339306258	3.40770033	0.000391111	**M13**	0.256147782	3.681042239	0.000208429
**IP12**	0.572629343	2.640767351	0.002286824	-	-	-	-

**Table 9:** FPS and FFP values of the basic events in the barrier failure fault tree based on IT2FS.

	FPS	K	FFP		FPS	K	FFP
**R01**	0.624366273	2.47070806	0.003382922	**IP13**	0.644209726	2.405482632	0.00393113
**R02**	0.681113739	2.284179138	0.005197816	**IP14**	0.517680591	2.821383896	0.001508746
**R03**	0.638057337	2.425705533	0.003752273	**IP15**	0.296639874	3.547944733	0.000283175
**R04**	0.67008858	2.320418837	0.004781687	**IP16**	0.695154045	2.238028653	0.005780579
**R05**	0.600977764	2.54758609	0.002834092	**IP17**	0.646428431	2.398189749	0.0039977
**R06**	0.74972593	2.058650867	0.008736734	**IP18**	0.60422444	2.536914266	0.002904596
**R07**	0.644511802	2.404489705	0.003940128	**IP19**	0.673973691	2.307648477	0.00492438
**R08**	0.637663487	2.427000117	0.003741105	**IP20**	0.638780159	2.423329618	0.003772857
**R09**	0.603519682	2.539230804	0.002889144	**E01**	0.593628241	2.571743972	0.002680748
**R10**	0.624251421	2.471085579	0.003379982	**E02**	0.280404978	3.601308837	0.000250433
**R11**	0.635179405	2.435165296	0.003671425	**E03**	0.559204073	2.684896212	0.002065874
**R12**	0.690717539	2.25261145	0.005589701	**E04**	0.672893861	2.311197879	0.004884298
**R13**	0.572969771	2.639648363	0.002292723	**E05**	0.407991274	3.181932682	0.00065776
**R14**	0.596429655	2.562535725	0.002738194	**E06**	0.583890507	2.603751904	0.00249028
**R15**	0.531288945	2.776653236	0.001672425	**E07**	0.591298982	2.579400245	0.002633903
**R16**	0.427616116	3.117425827	0.000763087	**E08**	0.655776886	2.367461375	0.004290803
**R17**	0.465247869	2.993730256	0.001014541	**E09**	0.540051701	2.747850058	0.001787104
**R18**	0.583978475	2.603462754	0.002491938	**E10**	0.559292074	2.684606954	0.00206725
**R19**	0.655776886	2.367461375	0.004290803	**E11**	0.684231035	2.273932586	0.005321909
**R20**	0.684231035	2.273932586	0.005321909	**E12**	0.682806709	2.278614348	0.005264846
**R21**	0.540051701	2.747850058	0.001787104	**E13**	0.619584061	2.486427193	0.003262667
**R22**	0.531984812	2.774365922	0.001681257	**E14**	0.656137034	2.36627757	0.004302515
**R23**	0.462629915	3.002335468	0.000994637	**E15**	0.586835994	2.594070088	0.002546419
**R24**	0.597283726	2.559728392	0.002755952	**E16**	0.480992679	2.941977064	0.001142939
**R25**	0.420716211	3.140105814	0.000724259	**E17**	0.339907031	3.405725589	0.000392893
**R26**	0.621190788	2.48114588	0.003302586	**E18**	0.681833413	2.281813571	0.005226205
**R27**	0.472857821	2.968716342	0.001074691	**E19**	0.441182741	3.07283233	0.000845605
**R28**	0.562005713	2.675687223	0.002110147	**E20**	0.56022143	2.681552159	0.002081842
**R29**	0.533856478	2.768213756	0.001705243	**E21**	0.604774412	2.535106509	0.002916712
**R30**	0.434957598	3.093294377	0.000806688	**E22**	0.465247869	2.993730256	0.001014541
**R31**	0.477340644	2.953981304	0.00111178	**E23**	0.458977717	3.014340244	0.00096752
**R32**	0.270506293	3.633845815	0.000232356	**E24**	0.646363521	2.398403107	0.003995737
**R33**	0.502297028	2.871949668	0.001342921	**E25**	0.591827608	2.577662652	0.002644462
**R34**	0.199557654	4.001001158	9.97697E-05	**E26**	0.492309524	2.904778595	0.001245149
**R35**	0.569371304	2.651476524	0.002231123	**H01**	0.281481973	3.597768755	0.000252482
**R36**	0.493272355	2.901613768	0.001254256	**H02**	0.435799566	3.090526826	0.000811845
**R37**	0.389738145	3.241930719	0.000572887	**H03**	0.633828423	2.439605975	0.003634076
**R38**	0.476586635	2.956459731	0.001105453	**H04**	0.519245567	2.81623982	0.001526723
**R39**	0.406817262	3.185791661	0.000651941	**H05**	0.361925185	3.333351918	0.000464139
**R40**	0.752235008	2.05040353	0.008904232	**H06**	0.435799566	3.090526826	0.000811845
**R41**	0.571969343	2.642936769	0.002275429	**H07**	0.563913755	2.669415487	0.002140841
**R42**	0.448578458	3.048522608	0.000894288	**H08**	0.64082051	2.416622984	0.003831572
**D01**	0.48739475	2.920933458	0.001199683	**H09**	0.581471024	2.611704745	0.002445092
**D02**	0.529698391	2.78188139	0.001652413	**H10**	0.481327259	2.940877299	0.001145837
**D03**	0.642904365	2.409773352	0.003892482	**H11**	0.421049345	3.139010804	0.000726088
**D04**	0.660359011	2.35239993	0.00444222	**H12**	0.49530301	2.894939006	0.001273682
**D05**	0.50580921	2.860405128	0.001379097	**H13**	0.494900072	2.896263463	0.001269804
**D06**	0.460371278	3.009759608	0.000977778	**H14**	0.464788035	2.99524173	0.001011017
**D07**	0.281745079	3.596903927	0.000252986	**H15**	0.437740155	3.084148112	0.000823857
**D08**	0.53615039	2.760673669	0.001735107	**H16**	0.572969771	2.639648363	0.002292723
**D09**	0.515008148	2.830168218	0.001478536	**H17**	0.528359526	2.786282237	0.001635753
**D10**	0.682806709	2.278614348	0.005264846	**H18**	0.467844013	2.985196728	0.001034673
**D11**	0.559204073	2.684896212	0.002065874	**H19**	0.604447367	2.536181506	0.002909501
**D12**	0.47870759	2.949488153	0.001123342	**H20**	0.434399563	3.095128638	0.000803288
**D13**	0.565789332	2.663250465	0.002171449	**H21**	0.477487918	2.953497214	0.00111302
**D14**	0.60564765	2.532236174	0.002936053	**H22**	0.49190106	2.906121217	0.001241306
**D15**	0.446265166	3.056126399	0.000878767	**M01**	0.484819567	2.929398084	0.001176527
**D16**	0.639184975	2.421998987	0.003784435	**M02**	0.461816965	3.005007636	0.000988536
**IP01**	0.27876954	3.606684522	0.000247352	**M03**	0.56465901	2.666965833	0.002152951
**IP02**	0.435799566	3.090526826	0.000811845	**M04**	0.428229922	3.115408246	0.00076664
**IP03**	0.301374637	3.532381568	0.000293507	**M05**	0.525740614	2.794890602	0.001603649
**IP04**	0.464788035	2.99524173	0.001011017	**M06**	0.491283496	2.908151149	0.001235517
**IP05**	0.193026964	4.024991208	9.4408E-05	**M07**	0.5704298	2.647997246	0.002249069
**IP06**	0.672893861	2.311197879	0.004884298	**M08**	0.550884121	2.712243893	0.001939796
**IP07**	0.424950509	3.126187676	0.000747846	**M09**	0.517164951	2.823078805	0.001502869
**IP08**	0.559204073	2.684896212	0.002065874	**M10**	0.545728883	2.729189161	0.001865567
**IP09**	0.37587921	3.287485038	0.00051584	**M11**	0.347453259	3.380921137	0.000415986
**IP10**	0.569883691	2.649792307	0.002239792	**M12**	0.600188128	2.550181622	0.002817205
**IP11**	0.442602111	3.06816686	0.000854738	**M13**	0.343899117	3.392603602	0.000404945
**IP12**	0.715810895	2.170129588	0.006758813	**-**	-	-	-

**Fig 11:**
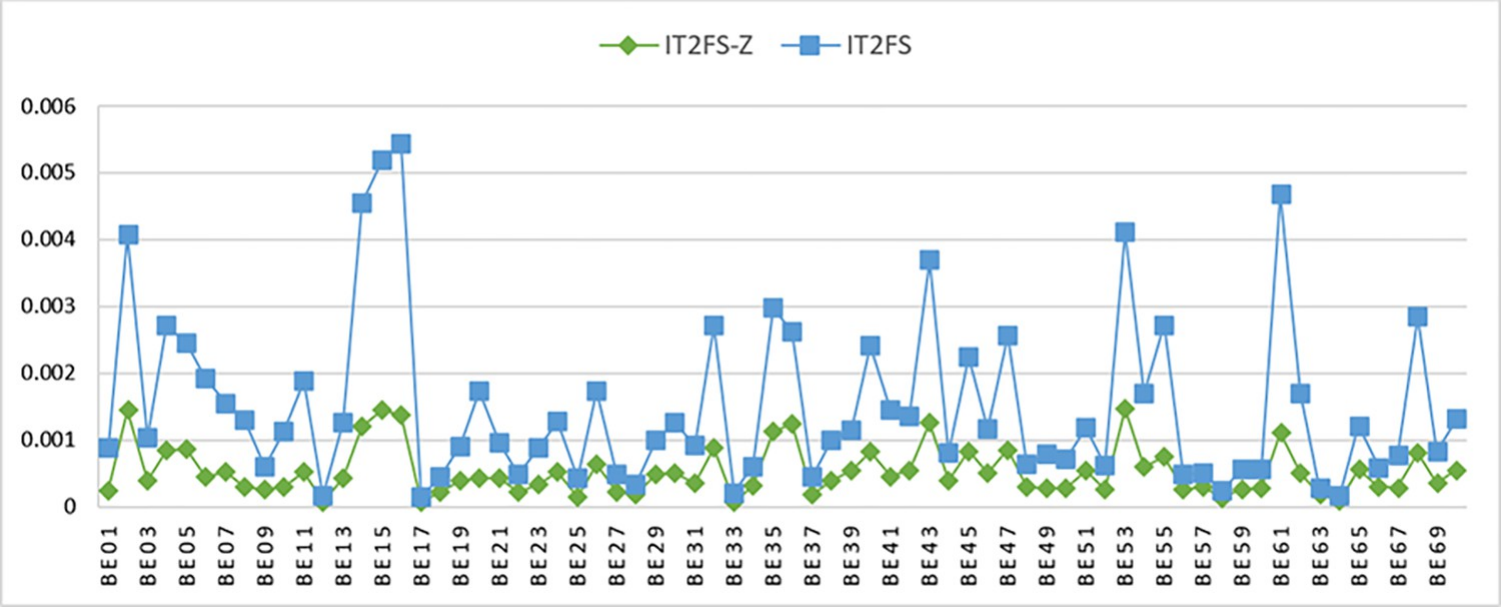
Comparison between probability of BEs calculated using IT2FS-Z and IT2FS in FTA.

These results are compared in Fig 11. The BEs prior probabilities in Fig 11 shows a significant difference in the results when combining opinion and confidence using the Z-number. The results obtained from IT2FS-Z are notably lower than those from IT2FS, consistent with Kang et al.’s study (2016) and Aghaei et al.’s (2021) [50, 61].

**Fig 12:**
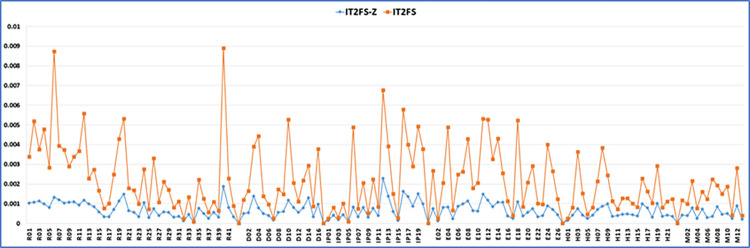
Comparison between probability of BEs calculated using IT2FS-Z and IT2FS in ETA.

Tables 8 and 9 show the prior probability of the BEs linked to the failure of RPB, DPB, IPB, and EPB barriers. The results of these tables were compared in Fig 12, which, like Fig 11, also shows the impact of using a z-number.

### 4.2. SHIPP concept

**Fig 13:**
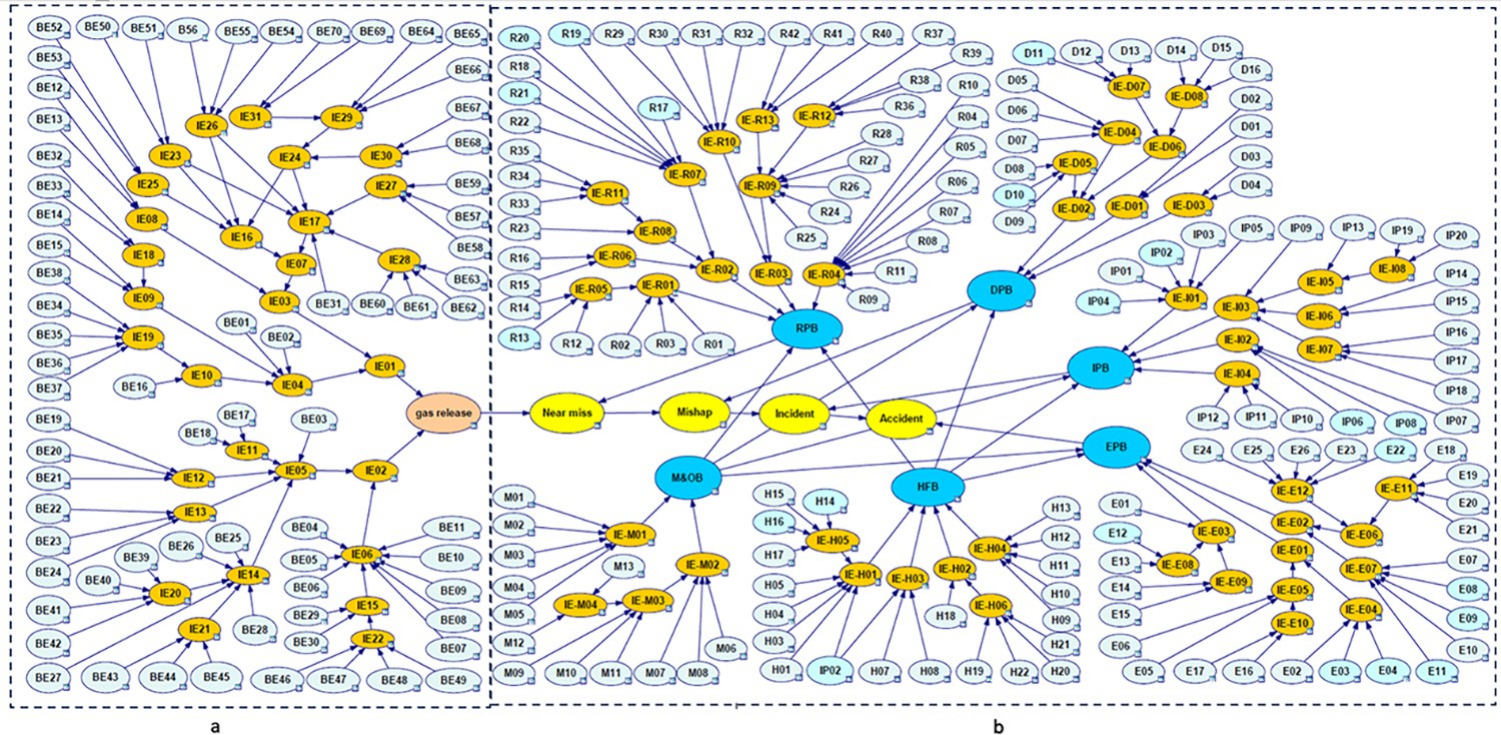
BN mapping from bow-tie (“a” is fault tree side of bowtie. “b” is event tree side with fault trees of barriers failure).

The SHIPP concept was applied in this study to ensure that obstacles and outcomes were logically connected to one another. Based on this approach, the barriers (RPB, DPB, IPB, EPB) that impact the probability of the consequences were identified on the bow-tie model’s event tree. In order to assess the failure probability of these barriers with a high level of confidence, a fault tree was created for each of these barriers (Figs 6–10 were mapped according to Fig 13 in the Bayesian network.) based on the Fuzzy probability of BEs.

One of the subtle and vital points in the SHIPP method is the relationship between HFB and M&OB with other obstacles and how they affect the results. Especially in BN, deciding whether HFB and M&OB, as a parent, are linked to other barriers and have an indirect effect on consequences or are directly linked to consequences can lead to different final values of the probability of consequences. It is also effective in detecting sensitive events to determine the posterior probability of barrier failure and consequences. So, based on the conceptual of SHIPP in the study by Samith Rathnayaka et al., who first used this method, HFB and M&OB in SHIPP have less of an effect on the outcomes than technical obstacles (RPB, DPB, IPB, EPB) [20]. Therefore, the impact of HFB and M&OB on outcomes was considered indirectly. For this purpose, HFB and M&OB were considered as parents and technical barriers as children in the BN. In the following, the technical barriers in the role of the parent were considered for the consequences, which can be observed in and the BN view in Fig 13.

### 4.3. Experts’ opinions

This study employed the opinions of twenty experts from the studied refinery. Experts expressed their opinions and confidence level according to the Linguistic terms in Table 1. These terms were quantified using the IT2FS (Eq 31) and combined using the Z-number (Eq 32) to reduce the uncertainty of the opinions.

### 4.4. Bayesian network

This section illustrates the bow-tie diagram of the spherical tank gas release shown in Fig 13, where part ’a’ represents the fault tree and part ’b’ is the event tree. In part b, the consequences and barriers are marked in yellow and blue, respectively. Also, in Fig 13, it is clear that each barrier has its fault tree.

**Fig 14:**
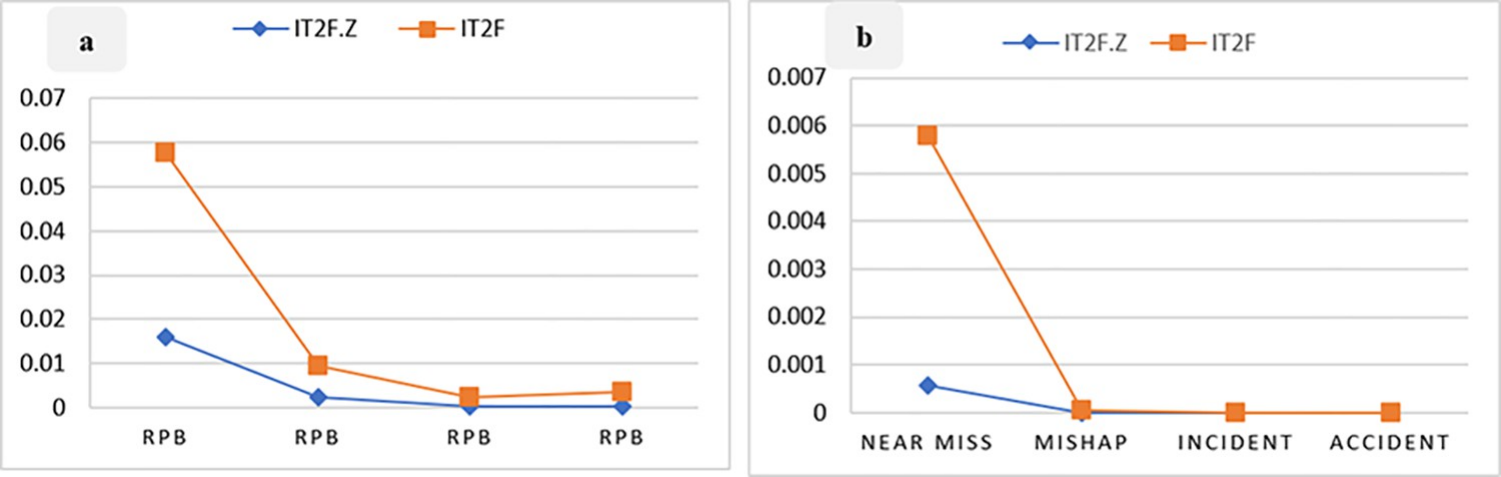
a: Prior probability of barrier failure. b: Prior probability of consequences.

Considering the prior probability of the BEs, the prior probability of barriers failure and consequences (Table 10) was calculated based on the Bayesian network conditional tables. The comparison of the prior probability of barrier failure in Fig 14 shows that the highest probability of failure is associated with the RPB, with 1.596 × 10^−2^ and 5.754 × 10^−2^) in IT2FS-Z and IT2FS, respectively. The highest probability for consequences is near miss, with probabilities of 5.764 × 10^−4^ in IT2FS-Z method and 5.784 × 10^−3^ in IT2FS. This Figure shows a decreasing trend in the probability of barriers and consequences in the subsequent layer, consistent with the study by Sarvestani et al. [2]. This can be due to the layer effect in the barriers. This means that by passing through each layer, the number of the next layer is reduced, and this lack of obstacles in each layer reduces the probability of their failure.

**Table 10:** Prior and probability of barriers failure and consequences based on BN.

barriers failure	RPB	DPB	IPB	EPB
**IT2FS-Z**	0.015962452	0.002327368	0.000269154	0.000349797
**IT2FS**	0.057542247	0.009504996	0.002328906	0.003462922
**consequences**	**Near miss**	**Mishap**	**Incident**	**Accident**
**IT2FS-Z**	0.000576444	1.38762E-06	2.34256E-09	4.34104E-11
**IT2FS**	0.005784105	5.81E-05	6.05E-07	3.89E-08

Fig 14 demonstrates the significant impact of employing Z-number to combine expert opinions with their confidence levels. Fig 14B shows that the trend of the plots obtained by the IT2FS-Z and IT2FS methods are indistinguishable for the other consequences, as the initial consequence (near miss) has a significantly higher prior probability than the subsequent consequences. Table 10 presents a more precise distinction between IT2FS-Z and IT2FS conditions.

#### 4.4.1. Sensitivity analysis in BN and identification of CBEs

**Fig 15:**
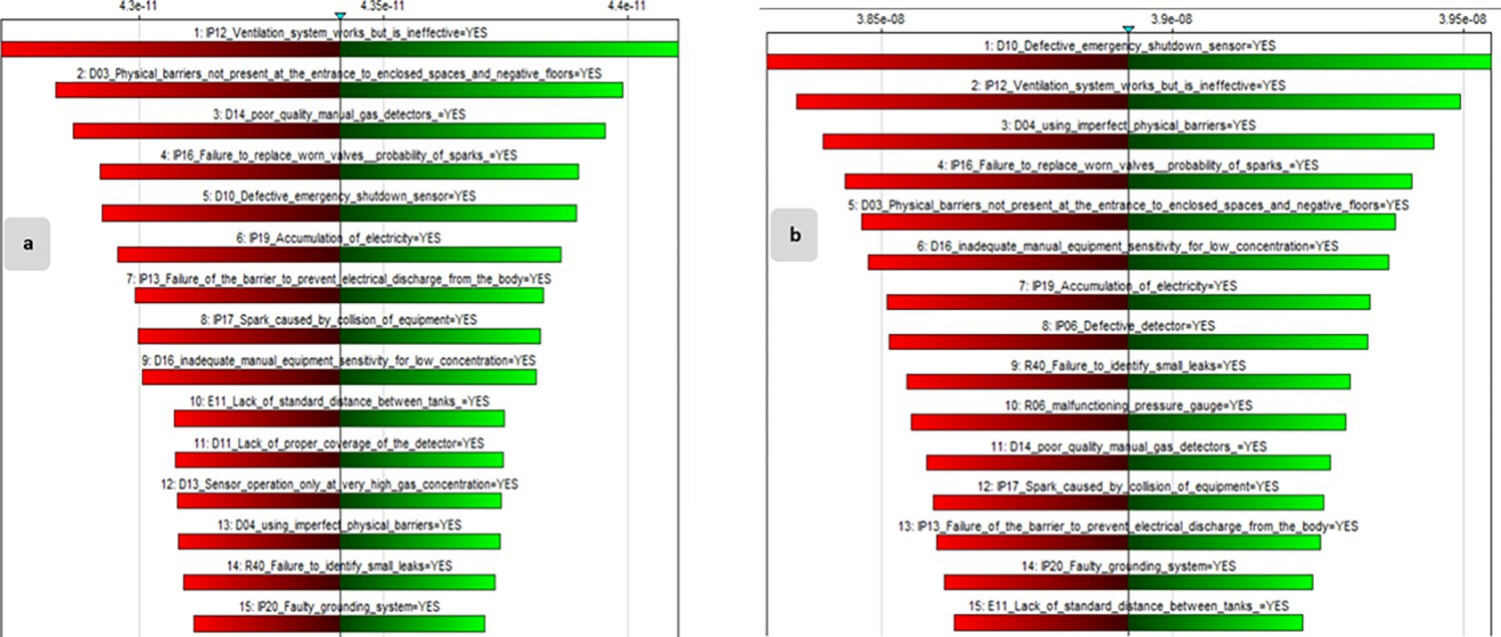
a: Tornado graph displaying the top fifteen CBEs in the IT2FS-Z BN. b: Tornado graph displaying the top fifteen CBEs in the IT2FS BN.

The explosion node was selected as the target node to determine the most critical BEs affecting the final consequence, and BN was used to do a sensitivity analysis. Then, fifteen events with the highest sensitivity were selected in the BN based on IT2FS-Z and IT2FS, depicted in Fig 15A and 15B, respectively. The probability values computed for the CBEs by the IT2FS-Z are different from those derived by the IT2FS technique, as shown by the Tornado diagram Fig 15A and 15B. Therefore, the rank of selected sensitive events using these two methods also differs. Therefore, IP12 (ventilation system works but is ineffective) with a prior probability of 2.286824 × 10^−3^ and D10 (defective emergency shutdown sensor) with a prior probability of 5.264846 × 10^−3^ are the most critical BEs in the tornado diagram derived from IT2FS-Z (Fig 15A) and IT2FS data (Fig 15B), respectively.

Furthermore, the BEs in the two fuzzy approaches have different prior probabilities. Thus, a CBE may be among the first fifteen priorities in one calculation mode, but it may be out of this prioritization in another method. For example, D11 (Insufficient detector coverage) ranks 11 in IT2FS-Z calculation method and 23 in IT2F, and D13 (Sensor operation only at very high gas concentration) ranks 12 in IT2FS-Z and 21 in IT2F. On the other hand, while R06 (malfunctioning pressure gauge) ranked 10th in terms of significance and impact on the top event in the IT2FS-based calculations, it did not make it into the top 15 CBEs in the IT2FS-Z calculations.

These differences highlight the importance of considering the confidence level using the Z-number. This is because using the Z-number changes the ranking of CBEs in terms of importance. By bringing the results closer to the real world, the Z-number will help industry experts plan and allocate resources more effectively, leading to the most significant possible reduction in accident-related costs.

Secondly, given this study’s ultimate goal of predicting the posterior probability of event occurrence and barrier failure, using z-numbers to make CBE prior probabilities more accurate and realistic leads to more accurate predictions and better results in beta distribution calculations.

Thirdly, this sensitivity analysis using a Bayesian network is a dynamic method that allows managers and experts to be aware of the essential CBEs at all times, providing them greater flexibility in short-term planning.

### 4.5. Probability adapting

Due to incomplete Accident documentation in the industry studied, several hypothetical demands and failures related to CBEs were considered for the last five years; this stage of the study was conducted by a brainstorming session with the consultation of experts. These events can be seen in Table 11.

**Table 11:** Five-year cumulative table of CBE-related failures and successes.

Rank in IT2FS	Rank in IT2FS-Z	BE	Basic event	First year		Second year		Third year		Fourth year		Fifth year	
				F*	S*	F	S	F	S	F	S	F	S
**2**	**1**	**IP12**	The ventilation system works but is ineffective	1	3	3	6	3	9	3	13	4	15
**5**	**2**	**D03**	Physical barriers not present at the entrance to enclosed spaces and underground floors	2	5	3	8	5	12	6	13	7	15
**11**	**3**	**D14**	poor quality manual gas detectors	1	2	3	4	3	6	5	8	7	12
**4**	**4**	**IP16**	Failure to replace worn valves (probability of sparks)	1	4	2	9	4	13	4	17	5	23
**1**	**5**	**D10**	The sensor connected to the emergency shutdown system failed	3	2	3	4	4	7	5	9	7	12
**7**	**6**	**IP19**	Accumulation of electricity	0	1	1	2	1	3	1	5	1	6
**13**	**7**	**IP13**	Failure of the barrier to prevent electrical discharge from the body	0	1	0	2	1	4	1	5	2	6
**12**	**8**	**IP17**	A spark caused by the collision of equipment	0	1	0	3	0	4	1	6	1	8
**6**	**9**	**D16**	inadequate manual equipment sensitivity for low concentration	2	3	3	6	6	9	7	12	9	15
**15**	**10**	**E11**	Lack of standard distance between tank	0	0	0	1	0	1	0	2	0	2
**23**	**11**	**D11**	Insufficient detector coverage	1	3	2	7	3	9	3	11	4	15
**21**	**12**	**D13**	Sensor operation only at a very high gas concentration	0	1	1	2	1	2	1	3	2	5
**3**	**13**	**D04**	using imperfect physical barriers	1	2	2	6	4	8	5	10	7	13
**9**	**14**	**R40**	Failure to identify small leaks	2	3	4	5	5	6	7	9	9	12
**14**	**15**	**IP20**	Faulty grounding system	1	2	1	3	1	4	1	5	2	6
**10**	**33**	**R06**	malfunctioning pressure gauge	0	0	1	2	1	5	2	6	2	7
**8**	**21**	**IP06**	Defective detector	0	1	0	3	1	4	2	6	2	9

*F: Failure S: Success

#### 4.5.1. Beta distribution

By determining the incidents related to the CBEs over five years and using the likelihood values obtained from the experts’ opinions as the prior probability, it was possible to calculate the posterior probability of the CBEs. Notably, to reduce the uncertainty more in the study and based on previous studies, the value of variances of 10^−4^ and 10^−5^ was considered for beta distribution calculations.

S2A and S2B Tables in S1 Table show the values of α (the number of failures in demand associated with the CBEs) and β (the number of successes) in IT2FS-Z and IT2FS for years zero to five with variances of 10^−4^ and 10^−5^. The likelihood obtained from the expert opinions is the probability of year zero.

Although the comparison between IT2FS-Z and IT2FS shows the difference in the posterior probabilities obtained, considering variances difference also helps reduce the uncertainty in the final results as much as possible. Ultimately, the posterior probabilities will be closer to reality with greater certainty.

#### 4.5.2. Sensitivity analysis of methodology

The sensitivity analysis validates and clarifies the performance of IT2FS and IT2FS-Z according to the information provided in Tables 12–14. The amount of variation of the posterior probability compared to the prior probability in different fuzzy states and different variances is comparable, which is explained in detail in sections 4.5.3 and 4.5.4. This can facilitate the determination of the worst case in posterior probability and the decision-making process by considering multiple aspects.

**Table 12:** The posterior probability of CBEs over the FIVE years.

	prior probability	VAR = 10^−4^					VAR = 10^−5^				
		First year	Second year	Third year	Fourth year	Fifth year	First year	Second year	Third year	Fourth year	Fifth year
IT2FS											
IP12	0.002286824	0.04408346	0.13142188	0.1727239	0.183338796	0.195637464	0.00663064	0.019137383	0.030547167	0.040434718	0.052386718
D03	0.001390649	0.11281403	0.18662898	0.23916455	0.272010468	0.291782898	0.015340573	0.034185022	0.06107532	0.088060031	0.113756605
D14	0.001308457	0.07773435	0.22226656	0.27987649	0.333147753	0.358332461	0.008951047	0.030732921	0.050251964	0.079191238	0.112317154
IP16	0.001617931	0.05643746	0.11138739	0.16664281	0.180277219	0.183866215	0.007703286	0.018893546	0.038712084	0.054518259	0.069924894
D10	0.001167649	0.22048468	0.32216133	0.36194565	0.3785015	0.388481133	0.02642714	0.049624566	0.076416524	0.104647475	0.136949891
IP19	0.001496035	0.00161167	0.0732425	0.12679481	0.15151251	0.161234129	0.001506186	0.008235502	0.014775807	0.0208705	0.026490083
IP13	0.001378566	0.00149572	0.00149572	0.06891199	0.107509816	0.162218095	0.001388727	0.001388727	0.008570227	0.015339291	0.028173141
IP17	0.001360256	0.00147768	0.00136026	0.00117372	0.051935998	0.075876964	0.001370419	0.001360256	0.001340375	0.008343295	0.014669506
D16	0.000966104	0.17236604	0.26852012	0.34779668	0.370148547	0.38227468	0.021237736	0.048260245	0.093592256	0.133505544	0.171995838
E11	0.001480922	0.0017322	0.00189277	0.00208617	0.002086165	0.002086165	0.001501366	0.001511802	0.001522383	0.001522383	0.001522383
D11	0.000805081	0.00093828	0.00080508	0.10012329	0.187335435	0.199872449	0.000815344	0.000805081	0.012905336	0.034643199	0.051968263
D13	0.000794556	0.20177722	0.35453251	0.42428038	0.450822563	0.458192377	0.025337568	0.068583753	0.113584627	0.162149987	0.207544865
D04	0.000793184	0.11265338	0.18872149	0.23164382	0.237451891	0.236784039	0.013233625	0.035093163	0.062331404	0.082943853	0.103457453
R40	0.00186703	0.00197926	0.05857102	0.10908922	0.146976542	0.196326863	0.001877157	0.0072621	0.012589249	0.017764713	0.027649031
IP20	0.000985	0.10250784	0.1899401	0.27123169	0.309181659	0.334423084	0.011137284	0.02965377	0.06202585	0.09494238	0.131331162
R06	0.001329669	0.00158838	0.09010782	0.13196713	0.188745955	0.212748857	0.001350159	0.008985674	0.01613796	0.029655759	0.041783836
IP06	0.000832765	0.12091466	0.19437572	0.2256708	0.231289322	0.257543408	0.012840955	0.024275653	0.034786967	0.044123034	0.061792438
**IT2FS-Z**											
IP12	0.002286824	0.02123795	0.05918929	0.08747626	0.105385157	0.124402012	0.008226109	0.012557568	0.016727552	0.020660102	0.025725646
D03	0.001390649	0.0501956	0.09941478	0.1519624	0.192746744	0.223053816	0.008948317	0.016233628	0.027674853	0.040453002	0.054127595
D14	0.001308457	0.03699538	0.1191275	0.17160825	0.23114626	0.275470236	0.006341986	0.01631128	0.02578096	0.040718442	0.05967536
IP16	0.001617931	0.02230372	0.04858126	0.08777159	0.110532425	0.127082046	0.007484464	0.010784081	0.017173517	0.023101556	0.029914701
D10	0.001167649	0.06015055	0.10561441	0.15021604	0.189999002	0.228716961	0.01094134	0.016491034	0.023627388	0.032176169	0.043522855
IP19	0.001496035	0.00502915	0.02575715	0.04472637	0.059931453	0.071801406	0.004934471	0.006969315	0.008977533	0.010925138	0.012815838
IP13	0.001378566	0.00403693	0.00403693	0.02863775	0.048690146	0.082740572	0.003941221	0.003941221	0.006458434	0.008914929	0.013750167
IP17	0.001360256	0.00410341	0.0039977	0.00380182	0.025212799	0.040804513	0.004007791	0.0039977	0.00397767	0.006402658	0.008725729
D16	0.000966104	0.05387486	0.11003787	0.18657759	0.236487949	0.274960306	0.009031367	0.016639384	0.031134432	0.046688612	0.064890997
E11	0.001480922	0.00553506	0.00564817	0.00576599	0.005765995	0.005765995	0.005342131	0.005352299	0.005362506	0.005362506	0.005362506
D11	0.000805081	0.00217685	0.00206587	0.0460082	0.106252368	0.133999257	0.002075993	0.002065874	0.006840083	0.015987234	0.024304629
D13	0.000794556	0.08640085	0.19711127	0.27843759	0.336235757	0.372173782	0.011287719	0.028662325	0.0488693	0.074267393	0.10261881
D04	0.000793184	0.0263575	0.06112058	0.09951029	0.123840092	0.144609851	0.006678246	0.011016409	0.017295267	0.023208374	0.03057775
R40	0.002286824	0.02123795	0.05918929	0.08747626	0.105385157	0.124402012	0.008226109	0.012557568	0.016727552	0.020660102	0.025725646
IP20	0.001390649	0.0501956	0.09941478	0.1519624	0.192746744	0.223053816	0.008948317	0.016233628	0.027674853	0.040453002	0.054127595
R06	0.001308457	0.03699538	0.1191275	0.17160825	0.23114626	0.275470236	0.006341986	0.01631128	0.02578096	0.040718442	0.05967536
IP06	0.001617931	0.02230372	0.04858126	0.08777159	0.110532425	0.127082046	0.007484464	0.010784081	0.017173517	0.023101556	0.029914701

**Table 13:** The posterior probability of barriers failure based on beta distribution and BN.

barriers	VAR = 10^−4^				VAR = 10^−5^			
	IT2FS-Z		IT2FS		IT2FS-Z		IT2FS	
	year	P*	year	p	year	p	year	p
**RPB**	1	0.015964	1	0.057746	1	0.015963	1	0.057562
	2	0.016948	2	0.069404	2	0.016056	2	0.058705
	3	0.017827	3	0.079747	3	0.016149	3	0.059809
	4	0.018485	4	0.098418	4	0.016239	4	0.061963
	5	0.018345	5	0.114643	5	0.016411	5	0.064122
**DPB**	1	0.22014	1	0.082387	1	0.029809	1	0.017437
	2	0.34896	2	0.167517	2	0.071173	2	0.030009
	3	0.424199	3	0.253018	3	0.124401	3	0.048789
	4	0.453603	4	0.311564	4	0.170009	4	0.068638
	5	0.471221	5	0.355438	5	0.212866	5	0.090842
**IPB**	1	0.003776	1	0.00617	1	0.000654	1	0.002727
	2	0.008097	2	0.012682	2	0.001545	2	0.003565
	3	0.011311	3	0.019993	3	0.002888	3	0.004887
	4	0.012751	4	0.025257	4	0.004169	4	0.006338
	5	0.013148	5	0.029933	5	0.005521	5	0.007935
**EPB**	1	0.000354	1	0.003473	1	0.00035	1	0.003464
	2	0.000357	2	0.003478	2	0.00035	2	0.003464
	3	0.00036	3	0.003484	3	0.000351	3	0.003465
	4	0.00036	4	3.48E-03	4	0.000351	4	0.003465
	5	0.000386	5	0.003484	5	0.000351	5	0.003465

*p: probability

**Table 14:** The posterior probability of consequences based on beta distribution and Bayesian network.

barriers	VAR = 10^−4^				VAR = 10^−5^			
	IT2FS-Z		IT2FS		IT2FS-Z		IT2FS	
	year	P*	year	p	year	p	year	p
**Near miss**	1	0.000577	1	0.005805	1	0.000576	1	0.005786
	2	0.000612	2	0.006976	2	0.00058	2	0.005901
	3	0.000644	3	0.008016	3	0.000583	3	0.006012
	4	0.000668	4	0.009893	4	0.000586	4	0.006229
	5	0.000662	5	0.011524	5	0.000593	5	0.006445
**Mishap**	1	0.000129	1	0.000497	1	1.76E-05	1	0.000106
	2	0.000234	2	0.001216	2	4.28E-05	2	0.000185
	3	0.000309	3	0.002104	3	7.59E-05	3	0.000306
	4	0.000349	4	0.003183	4	1.05E-04	4	0.000445
	5	0.000356	5	0.004232	5	1.35E-04	5	0.00061
**Incident**	1	2.02E-06	1	1.10E-05	1	6.04E-08	1	1.17E-06
	2	1.84E-05	2	5.38E-05	2	3.83E-07	2	2.54E-06
	3	4.51E-05	3	1.38E-04	3	1.35E-06	3	5.43E-06
	4	6.73E-05	4	2.48E-04	4	2.96E-06	4	1.01E-05
	5	6.70E-05	5	0.00039	5	5.84E-06	5	1.74E-05
**Accident**	1	3.77E-08	1	7.07E-07	1	1.12E-09	1	7.53E-08
	2	3.50E-07	2	3.47E-06	2	7.10E-09	2	1.64E-07
	3	8.68E-07	3	8.90E-06	3	2.51E-08	3	3.49E-07
	4	1.29E-06	4	1.60E-05	4	5.51E-08	4	6.50E-07
	5	1.38E-06	5	2.51E-05	5	1.09E-07	5	1.12E-06

* p: probability

#### 4.5.3. Posterior probability of the basic events

**Fig 16:**
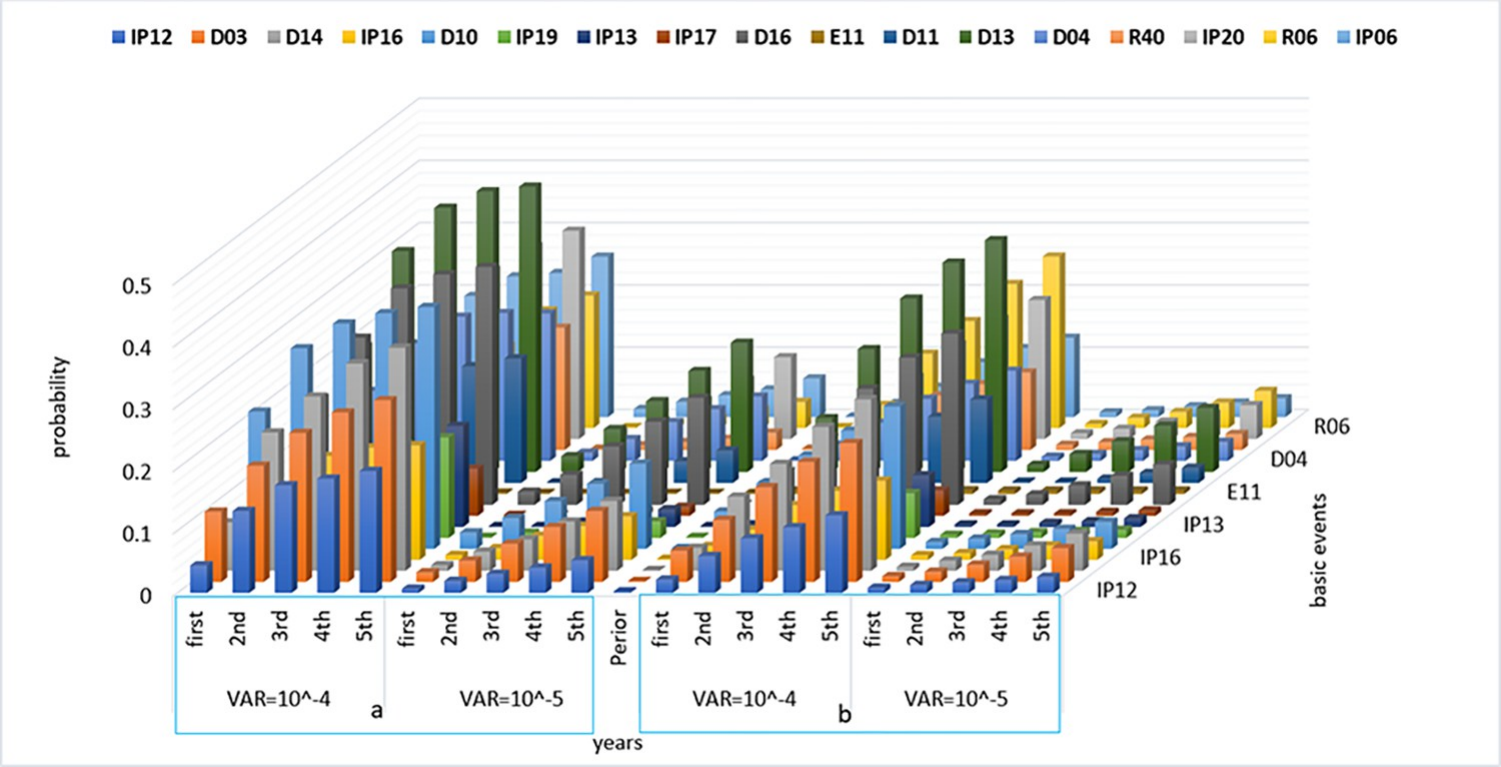
The prior and the posterior probability of the CBEs. (a: IT2FS. b: IT2FS-Z).

Calculating the posterior probability of CBEs for years zero to five (Table 12) based on the β and α values shows that the overall trend in the posterior probability of CBEs over five years is upward (Fig 16). This trend is consistent with the study by Ahmadi et al. [64].

According to Fig 16, The comparison of results reveals that incorporating Z-numbers to integrate the confidence levels in experts’ opinions reduces both prior and posterior probabilities (Fig 16B). This reduction aligns the results more closely with real-world scenarios, enhancing the model’s predictive accuracy and reliability. Notably, when comparing the fuzzy calculations in IT2FS and IT2FS-Z, utilizing Z-numbers results in lower probabilities for both prior and posterior assessments, indicating a more cautious and conservative approach to risk estimation.

Furthermore, the calculations based on the beta distribution for predicting future events, considering variances of 10^−4^ and 10^−5^, demonstrate that the variance of 10^−4^ signifies a higher probability of the event occurring in the future (Fig 16A and 16B). This distinction underscores the significance of selecting appropriate parameters, such as variance values, to improve the precision and robustness of risk assessments in dynamic environments.

Fig 16 visually represents these findings, illustrating the trend in posterior probabilities of CBEs over five years. The comparison between IT2FS and IT2FS-Z with variances of 10^−4^ and 10^−5^ showcases the impact of variance selection on the probability estimations. Specifically, the higher posterior probabilities observed with the variance of 10^−4^ in the IT2FS (Fig 16A) state highlight the increased confidence level in predicting critical events, while the lower probabilities associated with the variance of 10^−5^ in the IT2FS-Z model (Fig 16A) emphasize a more conservative risk assessment approach.

By leveraging these insights and understanding the impact of Z-numbers and variance selection on probability estimations, industry professionals can make more informed decisions, proactively address potential risks, and enhance safety measures to ensure operational resilience and efficiency in real-world applications.

#### 4.5.4. Posterior probability of barriers and consequences

**Fig 17:**
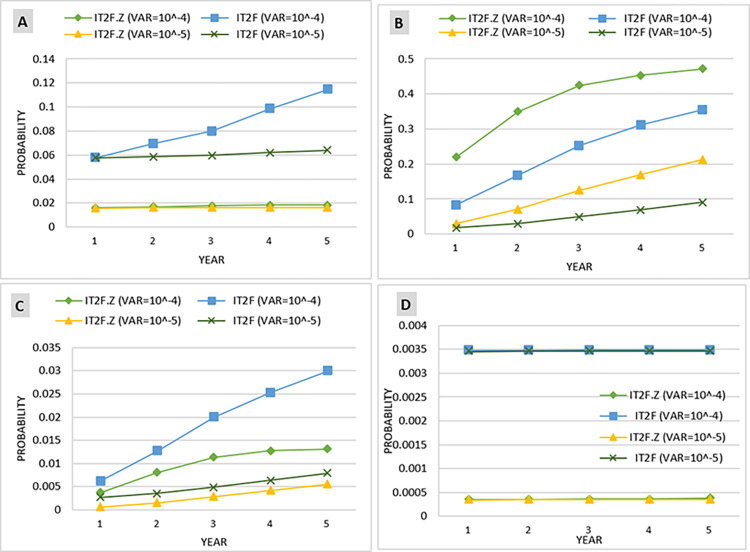
The posterior probability of barriers failure. (A: RPB. B: DPB. C: IPB. D: EPB).

The posterior probability of CBEs for years one to five were entered separately into the BN, and the posterior probability of barriers failure and consequences for each year were calculated by updating the Bayesian network. The results of calculating the posterior probability of barrier failure in different fuzzy states with variances of 10^−4^ and 10^−5^ are shown in Table 13. The results of this table were compared in Fig 17.

The overall comparison of the results in Fig 17 shows that combining Z-number with IT2F has led to significant changes in the posterior probability of barrier failure. In the IT2FS-Z mode, the posterior probability for both DPB (Fig 17B) and IPB (Fig 17C) has a significant difference in the variance of 10^−4^ compared to the variance of 10^−5^, while this difference is not as great for RPB (Fig 17D) and EPB (Fig 17A). This is due to the high number of CBEs present in the fault tree of DPB (seven CBEs) and IPB (six CBEs), which clarifies the effect of the posterior probability of these basic events in the respective barrier. While in the IT2F method, a significant difference in posterior probability can be seen in RPB, in addition to DPB and IPB, during the five years, which can be due to not considering the level of certainty of experts in their opinions.

This issue shows the importance of reducing the uncertainty in the determination of the CBEs and in the planning for the control and prevention of accidents involving spherical tanks.

**Fig 18:**
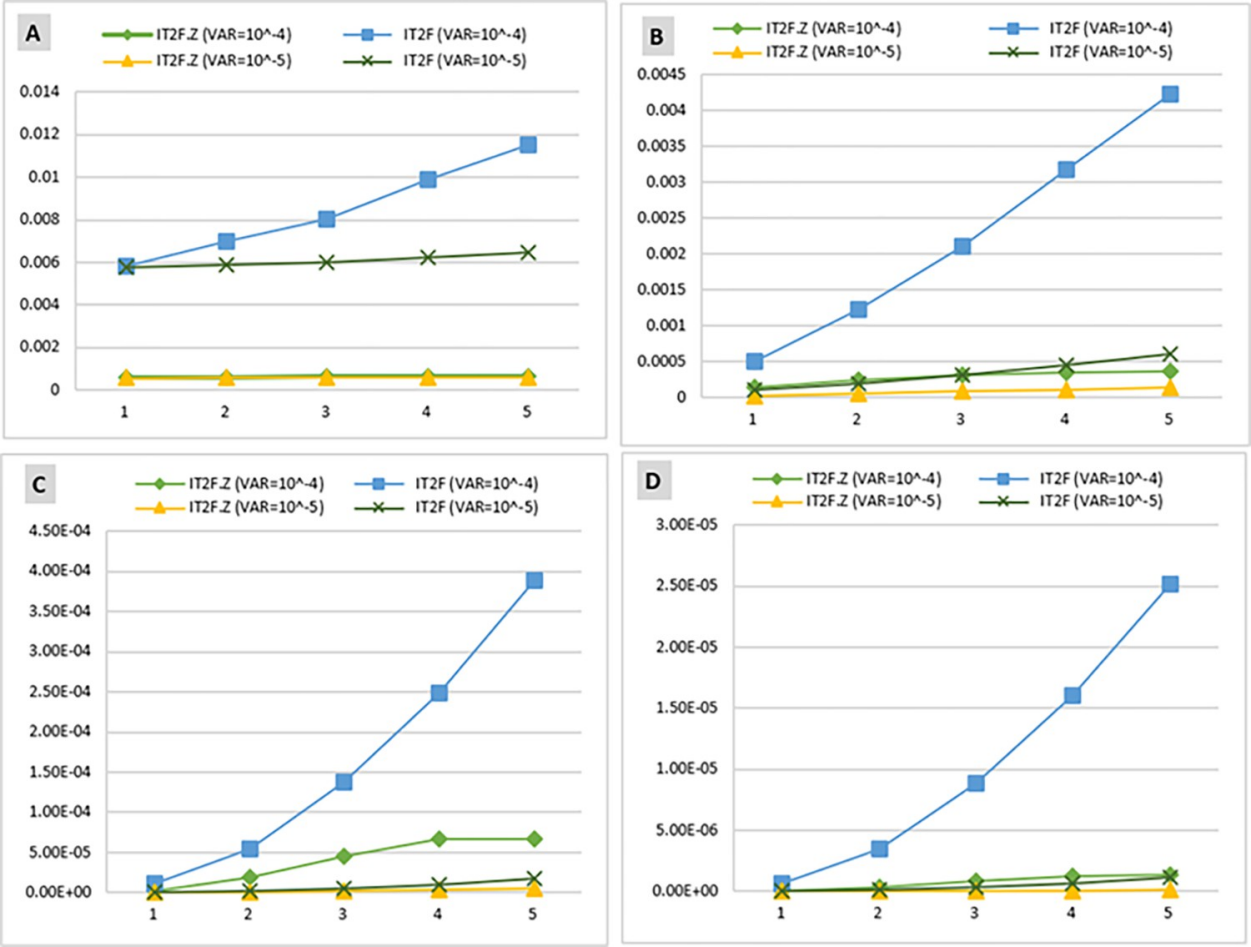
The posterior probability of consequences. (A: Near miss. B: mishap. C: Incident. D: Accident).

The results for the posterior probability of all four outcomes (A: near miss, B: mishap, C: incident, D: accident) are shown in Table 14. The results of this Table were compared in Fig 18. In the IT2F computation, the results obtained using the 10^−4^ variance, in comparison to those obtained using the 10^−5^ variance, indicate a worse case or the so-called higher posterior probability for barriers (Fig 18). This difference in the value of posterior probabilities increases towards the final years. This indicates the impact of various factors, such as wear and tear of equipment, on the increase in the probability and severity of accidents over time.

**Fig 19:**
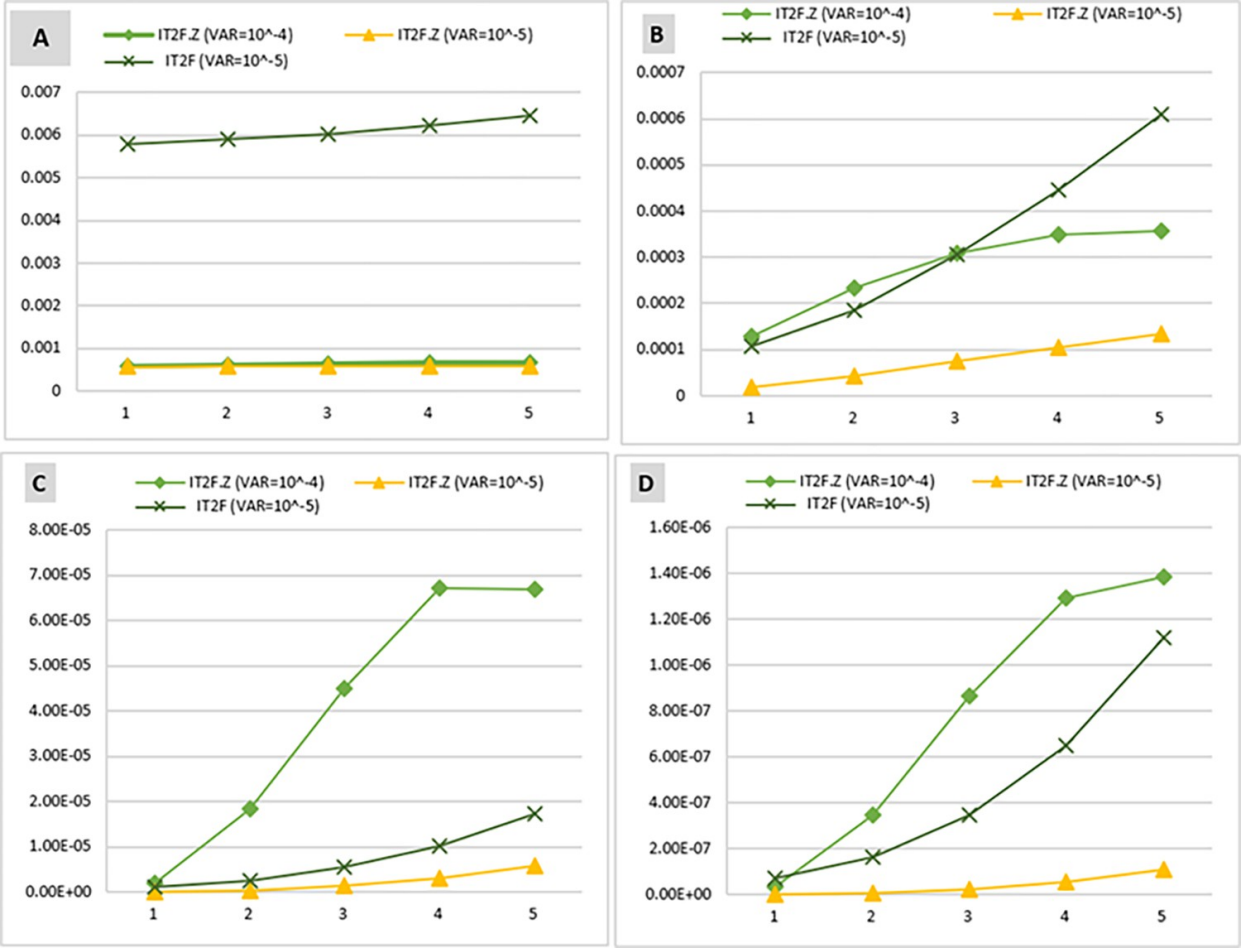
The posterior probability of the consequences in different calculations, except for IT2FS(VAR = 10^−4^) (A: Near miss. B: mishap. C: Incident. D: Accident).

For a better comparison of the results, in addition to Figs 18 and 19 has been drawn without the trend plot for near miss to show the difference between the plots for the other consequences. Accuracy in Fig 19 shows that plot ordering in Fig 19B (mishap) is not comparable to the scheme in Fig 17B (DPB). To explain this problem, it can be said that in addition to the DPB as a direct barrier, other barriers also indirectly affect the probability of the subsequent consequences. The same applies to the 19d (accident) and 17d (EPB) graphs.

In general, combining the experts’ confidence levels in their opinions using the Z-number has lowered the probability level in both prior and posterior cases compared to the point where only IT2FS was used. Yazdi et al. obtained results using type 1 fuzzy and Z-number in line with this study [60].

The trends in the posterior probability indicate that the method presented provides more reliable, realistic results over time. Irrespective of the difference between the different modes in the results, as seen in Figs 17–19. With this method’s continuous learning principles, the general trend in predicting future years’ probabilities is incremental. This dynamic prediction helps industries prioritize the necessary preventive measures by analyzing the results and spending the least to achieve the most significant benefit in reducing spherical tank accidents.

Integrating IT2FS, Z-numbers, and Bayesian networks offers a more sophisticated and comprehensive approach to dynamic risk assessments than traditional methods. The model can better handle uncertainties and provide more accurate risk predictions by incorporating fuzzy logic and expert opinions through Z-numbers. Traditional methods like FTA, ETA, and bowtie are widely used for risk assessment in various industries [25, 26]. While these methods provide a systematic approach to identifying potential failures and consequences, they may not effectively handle uncertainties and dynamic changes, while the introduced method Can overcome this limitation.

Another traditional method for risk assessment involves Monte Carlo simulation, which is useful for probabilistic analysis [70]. However, it may not capture the complexity of uncertainties and dependencies as comprehensively as the integrated IT2FS, Z-numbers, and Bayesian networks approach.

In conclusion, the integration of IT2FS, Z-numbers, and Bayesian networks not only improves the accuracy of dynamic risk assessments but also enhances the model’s real-world applicability by addressing uncertainties more effectively and providing valuable insights for decision-making in practical settings.

Despite these advantages, the present method does not separate the types of uncertainties. Future research could explore integrating techniques that account for both aleatory and epistemic uncertainties to enhance the current methodology and enable a more comprehensive risk assessment framework. Aleatory uncertainty is a natural variation, haphazardness, or incongruity of a physical system. uncertainty epistemic is based on ambiguity, vagueness, imperfection, ignorance, and deficiency in system behaviors [71]. This integration would enable a more comprehensive and robust risk assessment framework, capable of handling the complexities of both aleatory and epistemic uncertainties. By incorporating these advancements, researchers can refine risk assessment models, leading to more informed decision-making and improved safety outcomes in industrial settings.

Another limitation of this study is that implementing this model in practice may pose challenges, mainly due to the complexity involved in interpreting and propagating uncertainty through the network’s layers. Moreover, although powerful, the fuzzy logic components might require additional effort for some industry practitioners to grasp and utilize effectively. To address this limitation and make this methodology more accessible to industry experts, developing an application or software tool based on the fuzzy logic and Bayesian network approach utilized in this study is suggested. This software application can serve as a user-friendly platform for practitioners to input their data, visualize the risk assessment process, and better understand how uncertainty propagates through the network. Additionally, the software can include tutorials and training modules to assist users in learning and applying the methodology effectively within their work environment.

Furthermore, to enhance the usability of this proposed methodology, it is suggested to incorporate a case study, like this study’s case about spherical tanks, or practical examples that demonstrate the step-by-step application of the fuzzy logic and Bayesian network approach in real-world scenarios. The aim of these practical illustrations is to bridge the gap between theoretical complexity and practical implementation, making it easier for industry practitioners to grasp and utilize this methodology effectively.

## 5. Conclusions

This study presents a method for dynamic risk assessment of spherical tanks in a refinery industry using a combination of interval type 2 fuzzy and Z-number (IT2FS-Z) along with the beta distribution’s mean and the SHIPP concept. The first step was to draw a fault tree as part of a bow-tie diagram to identify the events leading to the top event. Then the possible consequences that the top event could cause were defined.

Based on the SHIPP concept, barriers were defined for each consequence, and the Fault Tree associated with failing each barrier was drawn.

Next, the quantitative probability of the base event in both the top event and barrier fault trees was determined using expert opinion. The IT2FS method was used to reduce the uncertainty of the judgments, and the Z-number was used to increase the confidence in the opinions to make the judgments as close to reality as possible. In addition, the power average operator weighting method was used to reduce the conflict between the experts’ opinions.

The bow-tie diagram was mapped into the Bayesian network following the previous steps. The first step was to identify the number of the fifteen most sensitive BEs related to the failure of the barriers in the Bayesian network to determine the posterior probability of consequences and the barrier failure. This part was done using data from IT2FS and IT2FS-Z computations to allow a comparison of the results from both.

Then, the number of demands and failures related to CBEs in the last five years were recorded, and their posterior probability was calculated using the mean of the beta distribution. The posterior probability of the barriers and consequences for five consecutive years was obtained by updating the BN based on the posterior probability of CBEs.

Based on the results obtained, the overall posterior probability of consequences shows an increasing trend over five years. Comparing the results from IT2FS-Z and IT2FS shows differences due to the effect of expert confidence in the IT2FS-Z method. These differences are more evident in the 10^−4^ variance than in the 10^−5^ variance.

Implementing the integrated IT2FS-Z and Bayesian network model in industrial settings holds significant potential to substantially improve workplace safety and operational efficiency. By utilizing this model for dynamic risk assessment, industry professionals stand to make significant strides in reducing accident rates, thereby fortifying safety protocols and mitigating operational risks. The practical implications of embracing this model encompass more precise risk predictions, early detection of potential hazards, and informed decision-making to prioritize preventive measures effectively. Ultimately, integrating IT2FS-Z and Bayesian networks presents a practical and dependable approach for industry professionals to elevate safety performance and optimize risk management strategies in real-world scenarios.

## Supporting information

S1 Appendix Calculate the basic event (IP12) probability by aggregating four experts’ opinions. (DOCX)

S1 Table Containing the following: S2A Table. α and β values were obtained based on IT2FS-Z for the first to fifth years. S2B Table. α and β values were obtained based on IT2FS for the first to fifth years.
(DOCX)
