# Supplementary material for: Process accident prediction using Bayesian network based on IT2Fs and Z-number: A case study of spherical tanks
Source: PLoS One. 2024 Aug 29;19(8):e0307883. doi: 10.1371/journal.pone.0307883 (PMC11361685; doi:10.1371/journal.pone.0307883)
Supplement: S1 Appendix — (DOCX) [file pone.0307883.s001.docx]

***S1 Appendix:* Calculation of the basic event (IP12) probability by aggregation four experts' opinions.**

| First Experts' opinions (possibility of BE) (Table1): | | E_1_: (VH): [(0.9, 1, 1, 1; 1), (0.95, 1, 1, 1; 0.9)]  E_2_: (VL): [(0, 0, 0, 0.1; 1), (0, 0, 0, 0.05; 0.9)]  E_3_: (MH): [(0.5, 0.7, 0.7, 0.9; 1), (0.6, 0.7, 0.7, 0.8; 0.9)]  E_4_: (MH): [(0.5, 0.7, 0.7, 0.9; 1), (0.6, 0.7, 0.7, 0.8; 0.9)] |
| --- | --- | --- |
| First Experts' confidence level (Table1): | | E_1_: (S): [(0.4, 0.6, 0.7, 0.9; 1), (0.5, 0.6, 0.7, 0.8; 0.9)]  E_2_: (S): [(0.4, 0.6, 0.7, 0.9; 1), (0.5, 0.6, 0.7, 0.8; 0.9)]  E_3_: (QS): [(0.1, 0.3, 0.4, 0.6; 1), (0.2, 0.3, 0.4, 0.5; 0.9)]  E_4_: (VC): [(0.7, 0.9, 1, 1; 1), (0.8, 0.95, 1, 1; 0.9)] |
| Converting the confidence level into the opinion (using Eq. 31) | | E_1_: $\frac{\left[ \frac{\left( 0.9-0.4 \right)+\left( 1\times0.6-0.4 \right)+\left( 1\times0.7-0.4 \right)}{4}+0.4 \right]+\left[ \frac{\left( 0.8-0.5 \right)+\left( 0.9\times0.6-0.5 \right)+\left( 0.9\times0.7-0.5 \right)}{4}+0.5 \right]}{2}$=0.6337  E_2_: 0.6338  E_3_: 0.3413  E_4_: 0.8943 |
| Adding weight of experts' confidence level to the opinions (using Eq.32) | | E_1_: $\sqrt{0.6337}\times$ [(0.9, 1, 1, 1; 1), (0.95, 1, 1, 1; 0.9)] =  [(0.7164, 0.796, 0.796, 0.796; 1), (0.756, 0.796, 0.796, 0.796; 0.9)]  E_2_: [(0, 0, 0, 0.0796; 1), (0, 0, 0, 0.0398; 0.9)]  E_3_: [(0.2921, 0.4089, 0.4089, 0.5257; 1), (0.3505, 0.4089, 0.4089, 0.4673; 0.9)]  E_4_: [(0.4728, 0.662, 0.662, 0.8511; 1), (0.5674, 0.662, 0.662, 0.7565; 0.9)] |
| Power average operator | (Using Eq.11) | E_1_:  $R_{d}\left( {\tilde{\tilde{A}}}_{1},\tilde{\tilde{1}} \right)=\frac{1}{2\times1\times0.9}\left[ 1\times\left( 0.796-0.796-0.796+0.796 \right) \right]-\frac{1}{2\times1\times0.9}\left[ 0.9\times(0.5\left( 0.796-0.756-0.796+0.7164 \right)-\left( 0.796-0.796-0.796+0.7164) \right) \right] +1-0.796- 0.5(0.7164-0.756+0.796-0.796)$ = 0.174  E_2_: $R_{d}\left( {\tilde{\tilde{A}}}_{2},\tilde{\tilde{1}} \right)$=0.957 E_3_: $R_{d}\left( {\tilde{\tilde{A}}}_{3},\tilde{\tilde{1}} \right)$=0.485 E_4_: $R_{d}\left( {\tilde{\tilde{A}}}_{4},\tilde{\tilde{1}} \right)$=0.167 |
|  | (Using Eq.10) | $d\left( {\tilde{\tilde{A}}}_{1}, {\tilde{\tilde{A}}}_{2} \right)=\left\vert R_{d}\left( {\tilde{\tilde{A}}}_{1},\tilde{\tilde{1}} \right)-R_{d}\left( {\tilde{\tilde{A}}}_{2},\tilde{\tilde{1}} \right) \right\vert$= 0.7839 $d\left( {\tilde{\tilde{A}}}_{1}, {\tilde{\tilde{A}}}_{3} \right)$= 0.3115 $d\left( {\tilde{\tilde{A}}}_{1}, {\tilde{\tilde{A}}}_{4} \right)$=0.0068  $d\left( {\tilde{\tilde{A}}}_{2}, {\tilde{\tilde{A}}}_{3} \right)$=0.4723 $d\left( {\tilde{\tilde{A}}}_{2}, {\tilde{\tilde{A}}}_{4} \right)$=0.7907  $d\left( {\tilde{\tilde{A}}}_{3}, {\tilde{\tilde{A}}}_{4} \right)$=0.3183 |
|  | (Using Eq.14) | $Sup\left( {\tilde{\tilde{A}}}_{1}, {\tilde{\tilde{A}}}_{2} \right)=1- d\left( {\tilde{\tilde{A}}}_{1}, {\tilde{\tilde{A}}}_{2} \right)$= 0.2161 $Sup\left( {\tilde{\tilde{A}}}_{1}, {\tilde{\tilde{A}}}_{3} \right)$=0.6885 $Sup\left( {\tilde{\tilde{A}}}_{1}, {\tilde{\tilde{A}}}_{4} \right)$=0.9932  $Sup\left( {\tilde{\tilde{A}}}_{2}, {\tilde{\tilde{A}}}_{3} \right)$=0.5277 $Sup\left( {\tilde{\tilde{A}}}_{2}, {\tilde{\tilde{A}}}_{4} \right)$=0.2093  $Sup\left( {\tilde{\tilde{A}}}_{3}, {\tilde{\tilde{A}}}_{4} \right)$=0.6817 |
|  | (Using Eq.13) | $T\left( {\tilde{\tilde{A}}}_{1} \right)$= $Sup\left( {\tilde{\tilde{A}}}_{1}, {\tilde{\tilde{A}}}_{2} \right)+ Sup\left( {\tilde{\tilde{A}}}_{1}, {\tilde{\tilde{A}}}_{3} \right)+ Sup\left( {\tilde{\tilde{A}}}_{1}, {\tilde{\tilde{A}}}_{4} \right)$*=1.8978*  $T\left( {\tilde{\tilde{A}}}_{2} \right)$= $Sup\left( {\tilde{\tilde{A}}}_{1}, {\tilde{\tilde{A}}}_{2} \right)$*+* $Sup\left( {\tilde{\tilde{A}}}_{2}, {\tilde{\tilde{A}}}_{3} \right)$*+* $Sup\left( {\tilde{\tilde{A}}}_{2}, {\tilde{\tilde{A}}}_{4} \right)$=0.9531  $T\left( {\tilde{\tilde{A}}}_{3} \right)$=$Sup\left( {\tilde{\tilde{A}}}_{1}, {\tilde{\tilde{A}}}_{3} \right)+ Sup\left( {\tilde{\tilde{A}}}_{2}, {\tilde{\tilde{A}}}_{3} \right)+Sup\left( {\tilde{\tilde{A}}}_{3}, {\tilde{\tilde{A}}}_{4} \right)$=1.8979 $T\left( {\tilde{\tilde{A}}}_{4} \right)$= $Sup\left( {\tilde{\tilde{A}}}_{1}, {\tilde{\tilde{A}}}_{4} \right)+ Sup\left( {\tilde{\tilde{A}}}_{2}, {\tilde{\tilde{A}}}_{4} \right)+Sup\left( {\tilde{\tilde{A}}}_{3}, {\tilde{\tilde{A}}}_{4} \right)=$*1.8842* |
|  | (Using Eq.30 and Table 3) | $\omega_{E1}$:0.03738 $\omega_{E2}$:0.0654 $\omega_{E3}$:0.042 $\omega_{E4}$:0.0467  $a_{2}^{U}$:$\frac{\left( 0.03738\times\left( 1+1.8978 \right)\times0.7164 \right)+\left( 0.0654\times\left( 1+0.9531 \right)\times0 \right)+\left( 0.042\times\left( 1+1.8979 \right)\times0.2921 \right)+\left( 0.467\times\left( 1+1.8842 \right)\times0.4728 \right)}{\left( 0.03738\times\left( 1+1.8978 \right) \right)+\left( 0.0654\times\left( 1+0.9531 \right) \right)+\left( 0.042\times\left( 1+1.8979 \right) \right)+\left( 0.467\times\left( 1+1.8842 \right) \right)}$=0.4554  $a_{2}^{U}$: $\frac{\left( 0.03738\times\left( 1+1.8978 \right)\times0.796 \right)+\left( 0.0654\times\left( 1+0.9531 \right)\times0 \right)+\left( 0.042\times\left( 1+1.8979 \right)\times0.4089 \right)+\left( 0.467\times\left( 1+1.8842 \right)\times0.662 \right)}{\left( 0.03738\times\left( 1+1.8978 \right) \right)+\left( 0.0654\times\left( 1+0.9531 \right) \right)+\left( 0.042\times\left( 1+1.8979 \right) \right)+\left( 0.467\times\left( 1+1.8842 \right) \right)}$=0.5920  $a_{3}^{U}$: $\frac{\left( 0.03738\times\left( 1+1.8978 \right)\times0.796 \right)+\left( 0.0654\times\left( 1+0.9531 \right)\times0 \right)+\left( 0.042\times\left( 1+1.8979 \right)\times0.4089 \right)+\left( 0.467\times\left( 1+1.8842 \right)\times0.662 \right)}{\left( 0.03738\times\left( 1+1.8978 \right) \right)+\left( 0.0654\times\left( 1+0.9531 \right) \right)+\left( 0.042\times\left( 1+1.8979 \right) \right)+\left( 0.467\times\left( 1+1.8842 \right) \right)}$=0.5920  $a_{4}^{U}$: $\frac{\left( 0.03738\times\left( 1+1.8978 \right)\times0.796 \right)+\left( 0.0654\times\left( 1+0.9531 \right)\times0.0796 \right)+\left( 0.042\times\left( 1+1.8979 \right)\times0.5257 \right)+\left( 0.467\times\left( 1+1.8842 \right)\times0.8511 \right)}{\left( 0.03738\times\left( 1+1.8978 \right) \right)+\left( 0.0654\times\left( 1+0.9531 \right) \right)+\left( 0.042\times\left( 1+1.8979 \right) \right)+\left( 0.467\times\left( 1+1.8842 \right) \right)}$=0.7040  $a_{1}^{L}$: $\frac{\left( 0.03738\times\left( 1+1.8978 \right)\times0.756 \right)+\left( 0.0654\times\left( 1+0.9531 \right)\times0 \right)+\left( 0.042\times\left( 1+1.8979 \right)\times0.3505 \right)+\left( 0.467\times\left( 1+1.8842 \right)\times0.5674 \right)}{\left( 0.03738\times\left( 1+1.8978 \right) \right)+\left( 0.0654\times\left( 1+0.9531 \right) \right)+\left( 0.042\times\left( 1+1.8979 \right) \right)+\left( 0.467\times\left( 1+1.8842 \right) \right)}$=0.5337  $a_{2}^{L}$: $\frac{\left( 0.03738\times\left( 1+1.8978 \right)\times0.796 \right)+\left( 0.0654\times\left( 1+0.9531 \right)\times0 \right)+\left( 0.042\times\left( 1+1.8979 \right)\times0.4089 \right)+\left( 0.467\times\left( 1+1.8842 \right)\times0.662 \right)}{\left( 0.03738\times\left( 1+1.8978 \right) \right)+\left( 0.0654\times\left( 1+0.9531 \right) \right)+\left( 0.042\times\left( 1+1.8979 \right) \right)+\left( 0.467\times\left( 1+1.8842 \right) \right)}$=0.5920  $a_{3}^{L}$: $\frac{\left( 0.03738\times\left( 1+1.8978 \right)\times0.796 \right)+\left( 0.0654\times\left( 1+0.9531 \right)\times0 \right)+\left( 0.042\times\left( 1+1.8979 \right)\times0.4089 \right)+\left( 0.467\times\left( 1+1.8842 \right)\times0.662 \right)}{\left( 0.03738\times\left( 1+1.8978 \right) \right)+\left( 0.0654\times\left( 1+0.9531 \right) \right)+\left( 0.042\times\left( 1+1.8979 \right) \right)+\left( 0.467\times\left( 1+1.8842 \right) \right)}$=0.5920  $a_{4}^{L}$: $\frac{\left( 0.03738\times\left( 1+1.8978 \right)\times0.796 \right)+\left( 0.0654\times\left( 1+0.9531 \right)\times0.0398 \right)+\left( 0.042\times\left( 1+1.8979 \right)\times0.4673 \right)+\left( 0.467\times\left( 1+1.8842 \right)\times0.7565 \right)}{\left( 0.03738\times\left( 1+1.8978 \right) \right)+\left( 0.0654\times\left( 1+0.9531 \right) \right)+\left( 0.042\times\left( 1+1.8979 \right) \right)+\left( 0.467\times\left( 1+1.8842 \right) \right)}$*=*0.6480  [(0.4554, 0.5920, 0.5920, 0.7040; 1), (0.5337, 0.5920, 0.5920, 0.6480; 0.9)] |
| Defuzzifying aggregated experts' opinions (using Eq.30) | | FPS:  $\frac{\left[ \frac{\left( 0.7040-0.4554 \right)+\left( 1\times0.5920-0.4554 \right)+(1\times0.5920-0.4554)}{4}+0.4554 \right]+\left[ \frac{\left( 0.6480-0.5337 \right)+\left( 0.9\times0.5920-0.5337 \right)+(0.90.5920-0.5337)}{4}+0.5337 \right]}{2}$  =0.5726 |
| calculating the probability of BE (using Eqs.33 and 35) | | 0.2$\leq$0.5726$\leq$ 0.8$\underset{\to}{Eq.35}$k = 4.523-3.287$\times$0.5726=2.6407  $FFP=\frac{1}{{10}^{2.6407}}=$0.002286 |
