# Supplementary material for: Process accident prediction using Bayesian network based on IT2Fs and Z-number: A case study of spherical tanks
Source: PLoS One. 2024 Aug 29;19(8):e0307883. doi: 10.1371/journal.pone.0307883 (PMC11361685; doi:10.1371/journal.pone.0307883)
Supplement: S1 Table — α and β values were obtained based on IT2FS-Z for the first to fifth years. S2B Table. α and β values were obtained based on IT2FS for the first to fifth years. (DOCX) [file pone.0307883.s002.docx]

**S2A Table. α and β values were obtained based on IT2FS-Z for the first to fifth years.**

| $\boldsymbol{\beta}_{\boldsymbol{5}}$ | $\boldsymbol{\alpha}_{\boldsymbol{5}}$ | $\boldsymbol{\beta}_{\boldsymbol{4}}$ | $\boldsymbol{\alpha}_{\boldsymbol{4}}$ | $\boldsymbol{\beta}_{\boldsymbol{3}}$ | $\boldsymbol{\alpha}_{\boldsymbol{3}}$ | $\boldsymbol{\beta}_{\boldsymbol{2}}$ | $\boldsymbol{\alpha}_{\boldsymbol{2}}$ | $\boldsymbol{\beta}_{\boldsymbol{1}}$ | $\boldsymbol{\alpha}_{\boldsymbol{1}}$ | $\boldsymbol{\beta}_{\boldsymbol{0}}$ | $\boldsymbol{\alpha}_{\boldsymbol{0}}$ |  | |
| --- | --- | --- | --- | --- | --- | --- | --- | --- | --- | --- | --- | --- | --- |
| VAR=$\boldsymbol{10}^{\boldsymbol{-4}}$ | | | | | | | | | | | | | |
| 57.7660551 | 14.0498892 | 44.7660551 | 10.0498892 | 33.7660551 | 7.04988923 | 26.7660551 | 4.04988923 | 22.7660551 | 1.04988923 | 21.7660551 | 0.04988923 | **IP12** |  |
| 55.8692295 | 23.0179215 | 42.8692295 | 16.0179215 | 31.8692295 | 10.0179215 | 21.8692295 | 5.0179215 | 15.8692295 | 2.0179215 | 12.8692295 | 0.0179215 | **D03** |  |
| 34.0516597 | 19.0157897 | 24.0516597 | 12.0157897 | 18.0516597 | 7.01578974 | 14.0516597 | 4.01578974 | 12.0516597 | 1.01578974 | 12.0516597 | 0.01578974 | **D14** |  |
| 71.1286163 | 16.0245167 | 50.1286163 | 11.0245167 | 35.1286163 | 7.02451672 | 24.1286163 | 3.02451672 | 17.1286163 | 1.02451672 | 15.1286163 | 0.02451672 | **IP16** |  |
| 34.6504055 | 22.0124505 | 24.6504055 | 15.0124505 | 17.6504055 | 10.0124505 | 12.6504055 | 6.01245047 | 10.6504055 | 3.01245047 | 10.6504055 | 0.01245047 | **D10** |  |
| 20.9171171 | 4.02085169 | 16.9171171 | 3.02085169 | 13.9171171 | 2.02085169 | 12.9171171 | 1.02085169 | 12.9171171 | 0.02085169 | 13.9171171 | 0.02085169 | **IP19** |  |
| 20.7490559 | 4.01759968 | 16.7490559 | 2.01759968 | 13.7490559 | 1.01759968 | 11.7490559 | 0.01759968 | 11.7490559 | 0.01759968 | 12.7490559 | 0.01759968 | **IP13** |  |
| 24.5669395 | 2.01711754 | 18.5669395 | 1.01711754 | 14.5669395 | 0.01711754 | 12.5669395 | 0.01711754 | 11.5669395 | 0.01711754 | 12.5669395 | 0.01711754 | **IP17** |  |
| 43.643348 | 27.0083584 | 30.643348 | 18.0083584 | 20.643348 | 11.0083584 | 13.643348 | 5.00835845 | 9.64334798 | 2.00835845 | 8.64334798 | 0.00835845 | **D16** |  |
| 9.7668708 | 0.0204179 | 9.7668708 | 0.0204179 | 9.7668708 | 0.0204179 | 10.7668708 | 0.0204179 | 11.7668708 | 0.0204179 | 13.7668708 | 0.0204179 | **E11** |  |
| 20.0386572 | 5.00567125 | 13.0386572 | 3.00567125 | 9.03865719 | 1.00567125 | 7.03865719 | 0.00567125 | 6.03865719 | 0.00567125 | 7.03865719 | 0.00567125 | **D11** |  |
| 31.9337332 | 27.0055136 | 21.9337332 | 18.0055136 | 14.9337332 | 11.0055136 | 10.9337332 | 6.00551362 | 7.93373319 | 2.00551362 | 6.93373319 | 0.00551362 | **D13** |  |
| 41.9200554 | 13.0054932 | 28.9200554 | 9.00549323 | 19.9200554 | 6.00549323 | 12.9200554 | 3.00549323 | 7.92005536 | 1.00549323 | 6.92005536 | 0.00549323 | **D04** |  |
| 20.6025161 | 5.0329259 | 17.6025161 | 3.0329259 | 16.6025161 | 2.0329259 | 16.6025161 | 1.0329259 | 16.6025161 | 0.0329259 | 17.6025161 | 0.0329259 | **R40** |  |
| 37.8315901 | 19.0087077 | 26.8315901 | 12.0087077 | 18.8315901 | 7.00870769 | 12.8315901 | 3.00870769 | 8.83159006 | 1.00870769 | 8.83159006 | 0.00870769 | **IP20** |  |
| 22.2626828 | 6.01632702 | 17.2626828 | 4.01632702 | 13.2626828 | 2.01632702 | 10.2626828 | 1.01632702 | 10.2626828 | 0.01632702 | 12.2626828 | 0.01632702 | **R06** |  |
| 17.3146186 | 6.00609644 | 13.3146186 | 4.00609644 | 10.3146186 | 3.00609644 | 8.31461859 | 2.00609644 | 7.31461859 | 1.00609644 | 7.31461859 | 0.00609644 | **IP06** |  |
| VAR=$\boldsymbol{10}^{\boldsymbol{-5}}$ | | | | | | | | | | | | |  |
| 262.6399699 | 14.51947367 | 249.6399699 | 10.51947367 | 238.6399699 | 7.519473667 | 231.6399699 | 4.519473667 | 227.6399699 | 1.519473667 | 226.6399699 | 0.519473667 | **IP12** |  |
| 180.6797787 | 23.19173088 | 167.6797787 | 16.19173088 | 156.6797787 | 10.19173088 | 146.6797787 | 5.191730877 | 140.6797787 | 2.191730877 | 137.6797787 | 0.191730877 | **D03** |  |
| 151.5048205 | 19.1696735 | 141.5048205 | 12.1696735 | 135.5048205 | 7.169673499 | 131.5048205 | 4.169673499 | 129.5048205 | 1.169673499 | 129.5048205 | 0.169673499 | **D14** |  |
| 216.2716013 | 16.25972862 | 195.2716013 | 11.25972862 | 180.2716013 | 7.259728615 | 169.2716013 | 3.259728615 | 162.2716013 | 1.259728615 | 160.2716013 | 0.259728615 | **IP16** |  |
| 139.493546 | 22.13501357 | 129.493546 | 15.13501357 | 122.493546 | 10.13501357 | 117.493546 | 6.135013572 | 115.493546 | 3.135013572 | 115.493546 | 0.135013572 | **D10** |  |
| 155.1577067 | 4.221981206 | 151.1577067 | 3.221981206 | 148.1577067 | 2.221981206 | 147.1577067 | 1.221981206 | 147.1577067 | 0.221981206 | 148.1577067 | 0.221981206 | **IP19** |  |
| 144.4781517 | 4.188403867 | 140.4781517 | 2.188403867 | 137.4781517 | 1.188403867 | 135.4781517 | 0.188403867 | 135.4781517 | 0.188403867 | 136.4781517 | 0.188403867 | **IP13** |  |
| 146.6571527 | 2.183417695 | 140.6571527 | 1.183417695 | 136.6571527 | 0.183417695 | 134.6571527 | 0.183417695 | 133.6571527 | 0.183417695 | 134.6571527 | 0.183417695 | **IP17** |  |
| 130.4247849 | 27.09227942 | 117.4247849 | 18.09227942 | 107.4247849 | 11.09227942 | 100.4247849 | 5.092279418 | 96.42478489 | 2.092279418 | 95.42478489 | 0.092279418 | **D16** |  |
| 142.6553797 | 0.21750729 | 142.6553797 | 0.21750729 | 142.6553797 | 0.21750729 | 143.6553797 | 0.21750729 | 144.6553797 | 0.21750729 | 146.6553797 | 0.21750729 | **E11** |  |
| 92.37932618 | 5.063958279 | 85.37932618 | 3.063958279 | 81.37932618 | 1.063958279 | 79.37932618 | 0.063958279 | 78.37932618 | 0.063958279 | 79.37932618 | 0.063958279 | **D11** |  |
| 103.3301809 | 27.06228721 | 93.33018087 | 18.06228721 | 86.33018087 | 11.06228721 | 82.33018087 | 6.062287206 | 79.33018087 | 2.062287206 | 78.33018087 | 0.062287206 | **D13** |  |
| 113.1934149 | 13.062071 | 100.1934149 | 9.062070999 | 91.19341491 | 6.062070999 | 84.19341491 | 3.062070999 | 79.19341491 | 1.062070999 | 78.19341491 | 0.062070999 | **D04** |  |
| 188.0083576 | 5.346062263 | 185.0083576 | 3.346062263 | 184.0083576 | 2.346062263 | 184.0083576 | 1.346062263 | 184.0083576 | 0.346062263 | 185.0083576 | 0.346062263 | **R40** |  |
| 126.3070356 | 19.09594193 | 115.3070356 | 12.09594193 | 107.3070356 | 7.095941933 | 101.3070356 | 3.095941933 | 97.30703557 | 1.095941933 | 97.30703557 | 0.095941933 | **IP20** |  |
| 141.6148608 | 6.175237208 | 136.6148608 | 4.175237208 | 132.6148608 | 2.175237208 | 129.6148608 | 1.175237208 | 129.6148608 | 0.175237208 | 131.6148608 | 0.175237208 | **R06** |  |
| 92.13869101 | 6.068459237 | 88.13869101 | 4.068459237 | 85.13869101 | 3.068459237 | 83.13869101 | 2.068459237 | 82.13869101 | 1.068459237 | 82.13869101 | 0.068459237 | **IP06** |  |

| $\boldsymbol{\beta}_{\boldsymbol{5}}$ | $\boldsymbol{\alpha}_{\boldsymbol{5}}$ | $\boldsymbol{\beta}_{\boldsymbol{4}}$ | $\boldsymbol{\alpha}_{\boldsymbol{4}}$ | $\boldsymbol{\beta}_{\boldsymbol{3}}$ | $\boldsymbol{\alpha}_{\boldsymbol{3}}$ | $\boldsymbol{\beta}_{\boldsymbol{2}}$ | $\boldsymbol{\alpha}_{\boldsymbol{2}}$ | $\boldsymbol{\beta}_{\boldsymbol{1}}$ | $\boldsymbol{\alpha}_{\boldsymbol{1}}$ | $\boldsymbol{\beta}_{\boldsymbol{0}}$ | $\boldsymbol{\alpha}_{\boldsymbol{0}}$ |  | |
| --- | --- | --- | --- | --- | --- | --- | --- | --- | --- | --- | --- | --- | --- |
| VAR=$\mathbf{10}^{\mathbf{-4}}$ | | | | | | | | | | | |  | |
| 101.684345 | 14.4469692 | 88.6843453 | 10.4469692 | 77.6843453 | 7.44696919 | 70.6843453 | 4.44696919 | 66.6843453 | 1.44696919 | 65.6843453 | 0.44696919 | **IP12** |  |
| 80.6262739 | 23.1470319 | 67.6262739 | 16.1470319 | 56.6262739 | 10.1470319 | 46.6262739 | 5.14703191 | 40.6262739 | 2.14703191 | 37.6262739 | 0.14703191 | **D03** |  |
| 50.191311 | 19.0830149 | 40.191311 | 12.0830149 | 34.191311 | 7.08301492 | 30.191311 | 4.08301492 | 28.191311 | 1.08301492 | 28.191311 | 0.08301492 | **D14** |  |
| 112.1452 | 16.3264388 | 91.1452003 | 11.3264388 | 76.1452003 | 7.32643877 | 65.1452003 | 3.32643877 | 58.1452003 | 1.32643877 | 56.1452003 | 0.32643877 | **IP16** |  |
| 75.1008121 | 22.2704618 | 65.1008121 | 15.2704618 | 58.1008121 | 10.2704618 | 53.1008121 | 6.27046185 | 51.1008121 | 3.27046185 | 51.1008121 | 0.27046185 | **D10** |  |
| 54.7649282 | 4.23637667 | 50.7649282 | 3.23637667 | 47.7649282 | 2.23637667 | 46.7649282 | 1.23637667 | 46.7649282 | 0.23637667 | 47.7649282 | 0.23637667 | **IP19** |  |
| 46.006763 | 4.14999919 | 42.006763 | 2.14999919 | 39.006763 | 1.14999919 | 37.006763 | 0.14999919 | 37.006763 | 0.14999919 | 38.006763 | 0.14999919 | **IP13** |  |
| 50.6620045 | 2.15517946 | 44.6620045 | 1.15517946 | 40.6620045 | 0.15517946 | 38.6620045 | 0.15517946 | 37.6620045 | 0.15517946 | 38.6620045 | 0.15517946 | **IP17** |  |
| 71.5622375 | 27.138893 | 58.5622375 | 18.138893 | 48.5622375 | 11.138893 | 41.5622375 | 5.13889304 | 37.5622375 | 2.13889304 | 36.5622375 | 0.13889304 | **D16** |  |
| 47.6594649 | 0.27639794 | 47.6594649 | 0.27639794 | 47.6594649 | 0.27639794 | 48.6594649 | 0.27639794 | 49.6594649 | 0.27639794 | 51.6594649 | 0.27639794 | **E11** |  |
| 32.5755373 | 5.04052431 | 25.5755373 | 3.04052431 | 21.5755373 | 1.04052431 | 19.5755373 | 0.04052431 | 18.5755373 | 0.04052431 | 19.5755373 | 0.04052431 | **D11** |  |
| 45.62246 | 27.0448781 | 35.62246 | 18.0448781 | 28.62246 | 11.0448781 | 24.62246 | 6.04487807 | 21.62246 | 2.04487807 | 20.62246 | 0.04487807 | **D13** |  |
| 78.0328524 | 13.1920144 | 65.0328524 | 9.19201437 | 56.0328524 | 6.19201437 | 49.0328524 | 3.19201437 | 44.0328524 | 1.19201437 | 43.0328524 | 0.19201437 | **D04** |  |
| 89.472577 | 5.77688949 | 86.472577 | 3.77688949 | 85.472577 | 2.77688949 | 85.472577 | 1.77688949 | 85.472577 | 0.77688949 | 86.472577 | 0.77688949 | **R40** |  |
| 65.4481909 | 19.1380346 | 54.4481909 | 12.1380346 | 46.4481909 | 7.1380346 | 40.4481909 | 3.1380346 | 36.4481909 | 1.1380346 | 36.4481909 | 0.1380346 | **IP20** |  |
| 94.8561351 | 6.74789968 | 89.8561351 | 4.74789968 | 85.8561351 | 2.74789968 | 82.8561351 | 1.74789968 | 82.8561351 | 0.74789968 | 84.8561351 | 0.74789968 | **R06** |  |
| 57.3719022 | 6.23251416 | 53.3719022 | 4.23251416 | 50.3719022 | 3.23251416 | 48.3719022 | 2.23251416 | 47.3719022 | 1.23251416 | 47.3719022 | 0.23251416 | **IP06** |  |
| VAR=$\boldsymbol{10}^{\boldsymbol{-5}}$ | | | | | | | | | | | | |  |
| 701.7826235 | 18.5305212 | 688.7826235 | 14.5305212 | 677.7826235 | 11.5305212 | 670.7826235 | 8.530521196 | 666.7826235 | 5.530521196 | 665.7826235 | 4.530521196 | **IP12** |  |
| 428.2277069 | 24.50535147 | 415.2277069 | 17.50535147 | 404.2277069 | 11.50535147 | 394.2277069 | 6.505351469 | 388.2277069 | 3.505351469 | 385.2277069 | 1.505351469 | **D03** |  |
| 312.8866856 | 19.85657367 | 302.8866856 | 12.85657367 | 296.8866856 | 7.856573672 | 292.8866856 | 4.856573672 | 290.8866856 | 1.856573672 | 290.8866856 | 0.856573672 | **D14** |  |
| 626.3999777 | 19.31641292 | 605.3999777 | 14.31641292 | 590.3999777 | 10.31641292 | 579.3999777 | 6.31641292 | 572.3999777 | 4.31641292 | 570.3999777 | 3.31641292 | **IP16** |  |
| 543.9607376 | 24.75200208 | 533.9607376 | 17.75200208 | 526.9607376 | 12.75200208 | 521.9607376 | 8.752002077 | 519.9607376 | 5.752002077 | 519.9607376 | 2.752002077 | **D10** |  |
| 493.6049621 | 6.408086074 | 489.6049621 | 5.408086074 | 486.6049621 | 4.408086074 | 485.6049621 | 3.408086074 | 485.6049621 | 2.408086074 | 486.6049621 | 2.408086074 | **IP19** |  |
| 397.0322496 | 5.535372095 | 393.0322496 | 3.535372095 | 390.0322496 | 2.535372095 | 388.0322496 | 1.535372095 | 388.0322496 | 1.535372095 | 389.0322496 | 1.535372095 | **IP13** |  |
| 407.5840656 | 3.587773863 | 401.5840656 | 2.587773863 | 397.5840656 | 1.587773863 | 395.5840656 | 1.587773863 | 394.5840656 | 1.587773863 | 395.5840656 | 1.587773863 | **IP17** |  |
| 409.5883148 | 28.42299034 | 396.5883148 | 19.42299034 | 386.5883148 | 12.42299034 | 379.5883148 | 6.422990344 | 375.5883148 | 3.422990344 | 374.5883148 | 1.422990344 | **D16** |  |
| 521.5467519 | 2.81187654 | 521.5467519 | 2.81187654 | 521.5467519 | 2.81187654 | 522.5467519 | 2.81187654 | 523.5467519 | 2.81187654 | 525.5467519 | 2.81187654 | **E11** |  |
| 217.7367805 | 5.423835983 | 210.7367805 | 3.423835983 | 206.7367805 | 1.423835983 | 204.7367805 | 0.423835983 | 203.7367805 | 0.423835983 | 204.7367805 | 0.423835983 | **D11** |  |
| 240.2050572 | 27.46832375 | 230.2050572 | 18.46832375 | 223.2050572 | 11.46832375 | 219.2050572 | 6.468323747 | 216.2050572 | 2.468323747 | 215.2050572 | 0.468323747 | **D13** |  |
| 474.2885445 | 14.96012366 | 461.2885445 | 10.96012366 | 452.2885445 | 7.960123659 | 445.2885445 | 4.960123659 | 440.2885445 | 2.960123659 | 439.2885445 | 1.960123659 | **D04** |  |
| 876.6456322 | 12.84903301 | 873.6456322 | 10.84903301 | 872.6456322 | 9.849033006 | 872.6456322 | 8.849033006 | 872.6456322 | 7.849033006 | 873.6456322 | 7.849033006 | **R40** |  |
| 402.4479533 | 20.41430168 | 391.4479533 | 13.41430168 | 383.4479533 | 8.414301683 | 377.4479533 | 4.414301683 | 373.4479533 | 2.414301683 | 373.4479533 | 1.414301683 | **IP20** |  |
| 867.4827205 | 13.55762742 | 862.4827205 | 11.55762742 | 858.4827205 | 9.557627419 | 855.4827205 | 8.557627419 | 855.4827205 | 7.557627419 | 857.4827205 | 7.557627419 | **R06** |  |
| 492.6750631 | 8.369100237 | 488.6750631 | 6.369100237 | 485.6750631 | 5.369100237 | 483.6750631 | 4.369100237 | 482.6750631 | 3.369100237 | 482.6750631 | 2.369100237 | **IP06** |  |

**S2B Table. α and β values were obtained based on IT2FS for the first to fifth years.**
